# Differential effects of fetal bovine serum and human platelet lysate on mesenchymal stromal cell-mediated support of hematopoietic stem/progenitor cells: a functional and transcriptomic analysis

**DOI:** 10.1186/s13287-025-04835-z

**Published:** 2025-11-29

**Authors:** Maria Catarina Carreira, André Branco, Miguel Casanova, Carolina Smet, Cláudia L. da Silva, Ana Fernandes-Platzgummer

**Affiliations:** 1 Department of Bioengineering and iBB, Institute for Bioengineering and Biosciences, Instituto Superior Técnico, Universidade de Lisboa, Lisbon, 1049-001 Portugal; 2 Associate Laboratory i4HB - Institute for Health and Bioeconomy at Instituto Superior Técnico, Universidade de Lisboa, Lisbon, 1049-001 Portugal; 3 Hospital São Francisco Xavier, Centro Hospitalar de Lisboa Ocidental, Lisbon, Portugal; 4 Present Address: GIMM, Lisbon, Portugal

**Keywords:** Umbilical cord blood, Hematopoietic stem/progenitor cells, Ex vivo expansion, Co-culture, Mesenchymal stromal cells, Human platelet lysate, Fetal bovine serum, Transcriptomic profiling

## Abstract

**Background:**

Umbilical cord blood (UCB) is a valuable source of hematopoietic stem and progenitor cells (HSPC) for transplantation. However, limited cell numbers require ex vivo expansion for effective treatment of adult patients. Mesenchymal stromal cell (MSC)-based co-cultures offer a supportive environment for HSPC expansion and a model to study niche interactions. Human platelet lysate (hPL) provides a xeno-free alternative to fetal bovine serum (FBS) for MSC culture, but its effect on MSC hematopoietic support remains unclear. This study investigates transcriptomic and functional changes induced by hPL-based culture and their impact on HSPC regulation.

**Methods:**

MSC from three bone marrow donors were expanded using hPL- or FBS-supplemented media under three regimens: continuously in cell isolation medium (Direct), adapted from the isolation medium to the other formulation (Adapted), or re-adapted back to the isolation medium (Re-adapted). Co-cultures with UCB-derived CD34^+^ HSPC assessed functional support over a 7-day period. Bulk transcriptomic profiling (RNA-seq) was performed on MSC under each condition. Differential gene expression and pathway enrichment (GO, KEGG) analysis characterized molecular differences and impacted signaling networks.

**Results:**

MSC properties were reversibly affected by the culture medium. Comparison of hPL-MSC and FBS-MSC revealed 13% differentially expressed genes (DEG), predominantly involved in extracellular matrix organization, chemokine signaling, and cell-cell communication. Minimal transcriptomic variation (1–2% DEG) was observed between *Direct* and *Adapted*/*Re-adapted* MSC. Co-culture with hPL-MSC resulted in significantly lower CD34^+^ cell expansion (2.4-fold reduction vs. FBS-MSC), though both outperformed the no feeder layer control. While proliferation was reduced, hPL-MSC promoted greater enrichment of primitive subsets, with increased CD34^+^CD45RA^−^ and CD34^+^CD45RA^−^CD90^+^ populations. Clonogenic potential remained comparable across all conditions. Network analysis identified dysregulation in TGF-β, PI3K-Akt, Notch, Wnt, and JAK/STAT pathways. hPL-MSC showed elevated expression of inhibitory and reduced expression of stimulatory hematopoietic regulatory factors.

**Conclusions:**

MSC cultured in hPL- or FBS-supplemented media display significant and reversible transcriptomic differences impacting HSPC expansion. Medium adaptation rapidly reprograms MSC phenotype and gene expression, highlighting responsiveness to environmental cues. Differential expression of key hematopoietic regulatory genes supports the observed functional disparities. These results provide a mechanistic basis for MSC-mediated HSPC support and lay groundwork for optimizing xeno-free expansion systems for clinical application.

**Supplementary Information:**

The online version contains supplementary material available at 10.1186/s13287-025-04835-z.

## Introduction

Umbilical cord blood (UCB) is an established source of hematopoietic stem and progenitor cells (HSPC) for hematopoietic cell transplantation (HCT). As a neonatal source of cells, UCB-derived HSPC (HSPC(CB)) tend to be more immature compared to adult bone marrow (BM) or mobilized peripheral blood, leading to a lower incidence of graft vs. host disease (GvHD), which allows a greater human leukocyte antigen (HLA) mismatch between donors and recipients [1]. Along with its biological potential, UCB has greater manufacturing leverage compared to adult sources, as it is easier to procure due to its non-invasive collection process. Another key advantage of UCB is its availability, since it is routinely discarded as medical waste, allowing the establishment of cryobanks with “off-the-shelf” UCB units [2]. However, due to inherently low collection volumes, the HSPC content is limited, and different strategies have been exploited to ensure successful cell engraftment and efficient hematopoietic reconstitution in adult patients, namely ex vivo HSPC expansion prior transplantation [3, 4].

Despite the significant advances in the field (recently reviewed in [5]), ex vivo HSPC expansion systems still require optimization to generate a fully expanded cell therapy product capable of supporting both long- and short-term hematopoietic recovery [6]. One of the most explored strategies is based solely on cytokine supplementation (i.e. liquid cultures), whose ubiquitous presence in HSPC expansion protocols highlights its importance [7]. In fact, cytokine optimization for ex vivo HSPC expansion has been previously pursued by our group [8, 9]. Nevertheless, even with cytokine cocktails, ex vivo expansion of HSPC in liquid suspension culture systems often result in limited cell proliferation and/or accelerated differentiation [8, 10]. Alternatively, a more directed approach based on Notch signaling has been extensively explored, using immobilized Notch ligands to induce Notch activation [11, 12]. Other approaches involve the use of biomaterials and/or incorporation of extracellular matrix (ECM) components to better mimic the structural, topographic, and non-cellular cues of the hematopoietic niche, leading to enhanced ex vivo HSPC expansion [13–15]. Additionally, several small molecules have been discovered to significantly improve HSPC expansion by promoting self-renewal instead of differentiation [16]. Well-known examples include StemRegenin-1 and UM171, which have been tested in phase I/II trials, including trials using a single expanded cord blood unit for HCT (StemRegenin-1 - NCT01930162, NCT03406962; UM171 - NCT02668315) [17, 18]. Notably, ex vivo expansion using the small molecule nicotinamide has reached phase III clinical trials (NCT02730299, NCT04260698), and was recently approved by the U.S. Food and Drug Administration (FDA) as the cell therapy Omisirge [19, 20].

Even though the aforementioned expansion systems have achieved early clinical success, they remain relatively simplistic and fail to replicate most interactions present in the bone marrow niche. This microenvironment is highly complex, containing diverse hematopoietic and non-hematopoietic cell populations, extracellular matrix components, and intricate signaling networks. This complexity makes it difficult to pinpoint the most critical interactions for HSPC regulation that could be exploited for ex vivo expansion [21]. To better mimic the niche, co-culture systems using supportive cells have been explored, namely using mesenchymal stromal cells (MSC), a key component of the hematopoietic microenvironment, known to regulate HSPC proliferation, differentiation, and self-renewal both in vivo and ex vivo [22, 23]. Despite the lack of knowledge regarding the mechanisms by which MSC support HSPC expansion, co-culture with MSC feeder layers (FL) have been shown to preserve hematopoietic stemness while enhancing proliferation [24–26]. Mechanistic studies have demonstrated that MSC support hematopoiesis via paracrine signaling mediated by cytokines, growth factors and extracellular vesicles (EVs), as well as through cell-cell contact [23–26]. Furthermore, there are some pathways that have been proven to play a role in MSC-HSPC interaction. Specifically, the Notch signaling pathway appears to have a strong influence, since modulating Notch ligand expression in MSC has been shown to impact their ability to prevent premature HSPC differentiation [27]. Other potentially relevant pathways include N-cadherin signaling, Wnt signaling, Tie2/Angiopoietin-1 signaling, and STAT signaling [28, 29]. Pinpointing the specific factors and pathway synergies responsible for mediating MSC support to HSPC remains a challenge due to the complexity and dynamic nature of the microenvironment, alongside with the significant cellular heterogeneity and overlapping signaling mechanisms. Nonetheless, HSPC expanded using MSC-based co-culture systems have already been studied in clinical trials (NCT00498316, NCT03096782), showing promising results with significant improvements in cell engraftment [30].

Although MSC-based co-culture represents a bio-inspired approach to recreate native cues from the hematopoietic niche, its use in a translational setting introduces additional complexity to the manufacturing of cell therapy products due to the inclusion of an extra cell type. MSC must be harvested from donor tissues and expanded using scalable, cost-effective, and reproducible platforms that comply with current good manufacturing practices (cGMP) [31]. The clinical translation of MSC expansion is often underestimated, since simple changes in what concerns culture conditions (e.g. expansion medium) can ultimately impact MSC functionality [32]. Traditionally, MSC expansion has relied on fetal bovine serum (FBS)-supplemented medium [33]. However, to produce cell-based therapies, it is important to guarantee that every step of the manufacturing process complies with cGMP [31]. While some clinical trials still use FBS-expanded MSC (e.g. NCT03326505 for the treatment of Multiple Sclerosis or NCT03706482 for Osteogenesis Imperfecta), there are concerns regarding the use of animal-derived products, as they pose safety risks such as prion transmission and unwanted immune reactions [34]. Furthermore, reducing the use of animal-derived products is increasingly prioritized to align with ethical standards. Consequently, transitioning to xeno(geneic)-free conditions at all stages of cell therapy manufacturing remains a key objective [31, 35].

Several xeno-free alternatives have been developed for ex vivo MSC expansion, though some remain under development, while others are associated with high costs [35]. Human platelet lysate (hPL) has emerged as a promising, cost-effective, xeno-free alternative that is gaining increasing attention [36, 37]. Derived from expired platelet concentrates, hPL has been shown to maintain or even improve MSC characteristics, with widespread acceptance in pre-clinical research and growing use in clinical trials [37, 31]. Compared to MSC expanded in FBS-supplemented media, hPL supplementation has been associated with enhanced MSC proliferation [37]. Nevertheless, studies suggest that MSC expanded with hPL-supplemented media may exhibit altered patterns in what concerns differentiation potential, as well as immunomodulatory and angiogenic capabilities [37–39]. Of notice, in a previous study by our group, MSC adapted from FBS- to hPL-supplemented medium showed a reduced ability to support the ex vivo expansion of UCB-derived CD34^+^- enriched cells in a co-culture system [40].

In this systematic study, we aim to comprehensively investigate the impact of hPL supplementation during MSC culture on MSC-mediated HSPC expansion in a co-culture system. Specifically, our goal is to elucidate the mechanisms underlying the reported hematopoietic support provided by MSC. To this end, BM-derived MSC (MSC(M)) were first cultured in medium supplemented with either FBS or hPL and subsequently used to establish co-culture systems for HSPC expansion. Expanded HSPC were evaluated for proliferation, immunophenotype, and clonogenic potential. Bulk transcriptomic analysis of MSC was performed to identify specific genes and pathways associated with their hematopoietic support capacity.

## Materials and methods

### MSC isolation and expansion

MSC(M) isolation and characterization were performed according to protocols previously established by the Stem Cell Engineering Research Group (SCERG) at iBB [41]. Briefly, BM aspirates were obtained from healthy donors after informed consent at Instituto Português de Oncologia Francisco Gentil, Lisboa, Portugal. Low density BM mononuclear cells (MNC) were isolated using a Ficoll density gradient (GEHealthcare) (See Additional File 1, Supplementary Table 1 for material sources and references) and then washed twice in Dulbecco’s Modified Eagle’s Medium (DMEM) (Gibco) supplemented with 1% (v/v) Antibiotic-Antimycotic (A.A.) and either 10% (v/v) FBS-MSC qualified (Gibco) (DMEM-FBS) or 5% (v/v) hPL UltraGRO™-PURE Gamma Irradiated (kindly provided by AventaCell Biomedical Corp.) (DMEM-hPL). Cells were plated at a density of around 200 000 cells/cm² in the respective culture medium and incubated at 37 °C and 5% CO_2_ in a humidified atmosphere, with medium changes every 3 to 4 days. MSC(M) were selected based on adherence to plastic and expanded until 70–80% confluence. Cells were then characterized according to the International Society for Cell & Gene Therapy (ISCT) minimal criteria for MSC characterization [42], cryopreserved and stored at the SCERG-iBB cell bank until further use.

MSC(M) from three healthy donors were obtained from the SCERG-iBB cell bank. Cryopreserved MSC(M) were thawed using either DMEM-FBS or DMEM-hPL. MSC(M) were typically seeded at a cell density of 3 000 cells/cm^2^ (occasionally varied between 2 300 and 4 100 cells/cm^2^) and incubated at 37 °C and 5% CO_2_ in a humidified atmosphere. Upon reaching 80–90% confluence, MSC(M) were detached using either a solution of 0.05% (v/v) Trypsin (Gibco) supplemented with 1 mM ethylenediaminetetraacetic acid (EDTA) (Thermo Fisher Scientific) in phosphate-buffered saline (PBS) (Gibco) or with TrypLE™ Select 10x (Gibco) diluted 1:10 in PBS (a xeno-free alternative for cell culture using DMEM-hPL). During each passage, cell number was determined using the Trypan Blue (Gibco) exclusion method.

**Fig. 1:**
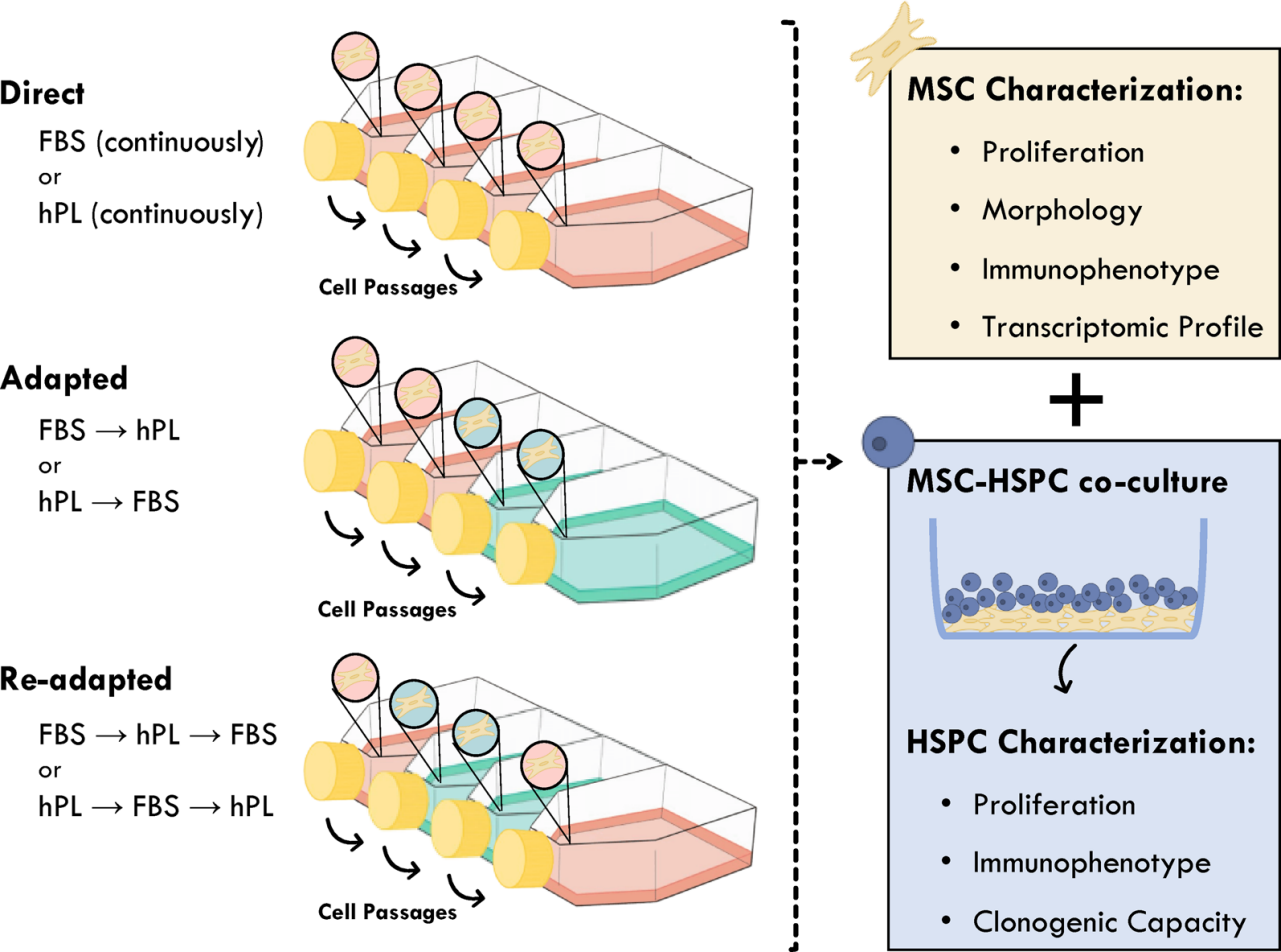
Experimental study design. Three different bone marrow-derived mesenchymal stromal cell (MSC(M)) donors were expanded in fetal bovine serum (FBS) or human platelet lysate (hPL)-supplemented expansion medium in standard tissue cultureware. Different adaptation regimens were used: (1) continuously in cell isolation medium (Direct), (2) adapted from the cell isolation medium to the other medium (Adapted), or (3) re-adapted back to the cell isolation medium (Re-adapted). During MSC expansion, cell proliferation, morphology, immunophenotype, and transcriptomic profile were assessed. Additionally, MSC from different conditions were used to establish feeder layers in co-culture systems for the expansion of hematopoietic stem/progenitor cells derived from the umbilical cord blood (HSPC(CB)). HSPC were expanded in co-culture with MSC for 7 days and cell proliferation, immunophenotype, and clonogenic capacity were assessed at the end of each expansion run

To establish the conditions for co-culture expansion with HSPC, MSC(M) underwent culture medium adaptation between DMEM-FBS and DMEM-hPL, using different approaches (Fig. 1). Upon isolation and subsequent MSC expansion, a fraction of cells was maintained in the same medium post-thawing (Direct condition). Two adaptation regimens, Adapted and Re-adapted, were introduced. For adaptation into hPL, MSC(M) initially isolated and expanded in DMEM-FBS were switched to DMEM-hPL (Adapted hPL). A fraction of these adapted MSC(M) was then switched back to DMEM-FBS after two passages (Re-adapted FBS). The same methodology was symmetrically applied, with MSC(M) initially isolated in DMEM-hPL being adapted to DMEM-FBS (Adapted FBS) and subsequently re-adapted to DMEM-hPL (Re-adapted hPL). In summary, MSC(M) were expanded under three experimental conditions: (1) continuously in cell isolation medium (Direct), (2) adapted from the cell isolation medium to the other medium (Adapted), or (3) re-adapted back to the cell isolation medium (Re-adapted).

### MSC immunophenotype

The expression of surface markers defined by the ISCT as minimal criteria for MSC characterization [42], along with other relevant markers, was assessed by flow cytometry. Briefly, detached MSC(M) were washed with PBS and incubated with LIVE/DEAD Fixable Far Red Dead Cell Stain (Invitrogen) (L/D) for 15 min in the dark to assess cell viability. After washing to remove excess dye, surface staining with anti-human monoclonal antibodies was performed with an incubation period of 15 min in the dark. The following antibodies were used: CD73 FITC [clone: AD2] (BD Biosciences), CD90 PE [clone: 5E10] (BioLegend), CD44 PerCP-Cy5.5 [clone: G44-26] (BD Biosciences), CD14 FITC [clone: M5E2] (BioLegend), CD105 PE [clone: 43A3] (BioLegend), CD34 PerCP-Cy5.5 [clone: 8G12] (BD Biosciences), HLA-DR FITC [clone: L243] (BioLegend), CD80 PE [clone: W17149D] (BioLegend), CD45 PerCP-Cy5.5 [clone: H130] (BioLegend), CD19 FITC [clone: HIB19] (BioLegend), CD11b PE [clone: ICRF44] (BioLegend), CD146 PE [clone: SHM-57] (BioLegend) and CD271 PE [clone: ME20.4] (BioLegend). Data acquisition was performed using a FACSCalibur cytometer (BD Biosciences) and data analysis was done using FlowJo V.10 software (FlowJo LLC).

### Bulk mRNA sequencing

Total RNA was extracted using a High Pure RNA Isolation Kit (Roche) according to the manufacturer’s instructions. Briefly, cells were resuspended in PBS and lysed by adding Lysis/-Binding Buffer, followed by centrifugation through a High Pure Filter Tube. After lysis, DNA contamination was removed by DNase I treatment, and subsequent washing steps were performed to increase purity. The isolated RNA was eluted with Elution Buffer and stored at −80 °C until further use. Library preparation and paired-end 150 bp sequencing were performed by Novogene (Beijing) using an Illumina NovaSeq 6000 platform. Transcriptomic data is available via GEO database (accession: GSE294580). Gene counts were normalized using the DESeq2 package and principal component analysis (PCA) was performed using the PCAtools package in R [43]. Differentially expressed genes (DEG) were defined as those with an adjusted p-value for multiple testing under 0.05 (padj < 0.05) and fold changes (FC) in expression greater than 2 (log_2_(FC) >1), determined with the DESeq function. Gene Set Enrichment Analysis (GSEA) was performed using the fgseaMultilevel function, by providing a list of all genes including their respective Walt statistic (stat) and a target gene set for which the enrichment score was calculated [44]. Specifically, Hallmark Pathways were used to perform GSEA [45]. A list of genes of interest (GOI) was defined having in mind experimentally validated cell phenotypes. DEG between Direct FBS and Direct hPL conditions that were consistently over- or underexpressed across all FBS vs. hPL conditions, with an error rate below 5%, were considered GOI. Gene Ontology (GO) analysis was performed using the enrichGO function [46]. After providing a list of GOI as input and using org.Hs.eg.db, “ALL” ontologies were selected. Furthermore, pathway enrichment analysis (PEA) was performed using the enrichr function for KEGG (KEGG_2021_Human), Hallmark (MSigDB_Hallmark_2020), Reactome (Reactome_2022), and WikiPathway (WikiPathway_2021_Human) [47]. Lastly, a list of 325 genes encoding factors known to impact the expansion of HSPC was constructed (Additional File 2). GSEA was performed using this list as the target gene set, and PEA was done for the genes in that list that were considered GOI or DEG.

### MSC-HSPC co-culture expansion

#### MSC feeder layer (FL) Preparation

MSC(M) previously expanded under the abovementioned conditions were plated onto 12-well plates. Once cells reached total confluence, MSC(M) growth was arrested by incubating the cells in their expansion medium supplemented with 0.5 µg/mL Mitomycin C (Sigma) for 3 h at 37 °C. Mitomycin C-treated feeder layers (FL) were then carefully washed twice and maintained in the respective expansion medium at 37 °C for up to one day until the beginning of the co-culture expansion.

#### Mononuclear cell (MNC) isolation

MNC from umbilical cord blood (MNC(CB)) were isolated using a Ficoll-Paque (GE Healthcare) density gradient-based phase separation. The layer containing MNC(CB) (i.e. MNC ring) was aspirated and washed with 2 mM EDTA in PBS. To eliminate any erythrocyte contamination, the cells obtained from each sample were treated with 40mL of ammonium chloride at 155 mM for 10 min at 4 °C. Isolated MNC(CB) were cryopreserved in DMEM supplemented with 10% FBS (Gibco) and 10% dimethyl sulfoxide (DMSO) (Sigma) and stored in a liquid/vapor phase nitrogen tank.

#### Establishment of a cryopreserved pool of CD34^+^-enriched cells

MNC(CB) from 3 different UCB donors were thawed and pooled for CD34^+^ cell enrichment using Magnetic Activated Cell Sorting (MACS) with the CD34 MicroBead Kit human (Miltenyi Biotec), according to the manufacturer’s instructions. A minimum sorting quality criterion of 70% CD34 expression was defined. After confirming CD34 expression by flow cytometry, the resulting pool of CD34^+^-enriched HSPC(CB) was cryopreserved as previously described for MNC(CB) and used throughout the study. Prior to cryopreservation, the enriched HSPC(CB) population was characterized as a non-expanded control (D0). An immunophenotypic analysis was performed by flow cytometry and the clonogenic capacity of the freshly sorted population was assessed through colony-forming unit (CFU) assay, as described in the “HSPC characterization” section.

#### Ex vivo expansion of HSPC

For the establishment of each co-culture system, the pool of CD34^+^-enriched cells was partially thawed. Recovered HSPC(CB) were seeded at a density of 60 000 cells/well (30 000 cells/mL) onto previously prepared growth-arrested MSC(M) FL. As an assay control, HSPC(CB) were also seeded into culture wells without a FL. HSPC(CB) were expanded in StemSpan Serum-Free Expansion Medium II (STEMCELL Technologies) supplemented with 1% A.A. and a previously optimized cytokine cocktail composed of stem cell factor (SCF − 90 ng/mL) (PeproTech), fms-like tyrosine kinase 3 ligand (Flt-3 L − 82 ng/mL) (PeproTech), thrombopoietin (TPO − 77 ng/mL) (PeproTech) and basic fibroblast growth factor (bFGF − 5 ng/mL) (PeproTech) for 7 days at 37 °C and 5% CO_2_ in a humidified atmosphere [8].

### HSPC characterization

#### Proliferation assay

At the end of each expansion run, expanded HSPC were harvested through forced pipetting and their total nucleated cell (TNC) number was determined. Cell viability was assessed through the Trypan Blue exclusion method. HSPC fold change (FC) was calculated as the ratio between TNC at the end of an expansion (D7) and the initially seeded cells (D0).

#### HSPC immunophenotype

HSPC immunophenotype was assessed, before and after expansion, by flow cytometry. After incubating with the L/D stain, surface staining was done as previously described for MSC, using the following anti-human monoclonal antibodies: CD45RA FITC [clone: HI100] (BD Biosciences), CD90 PE [clone: 5E10] (Biolegend) and CD34 PerCP-Cy5.5 [clone: 8G12] (BD Biosciences). Data acquisition was performed using a FACSCalibur Cytometer (BD Biosciences) and data analysis was performed using FlowJo V.10 software (FlowJo LLC).

#### In vitro clonogenic assay

To discern the clonogenic potential of non-expanded and expanded HSPC from the different co-culture systems, CFU assays were performed. 1 000 (before the expansion) or 2 500 (after the expansion) cells were carefully resuspended in semi-solid MethoCult H4434 Classic medium (STEMCELL Technologies) and seeded onto 3 wells of a 24-well plate. Cells were cultured at 37 °C and 5% CO_2_ in a humidified atmosphere for 14 days. At day 14, colonies were manually counted and classified as multilineage colony-forming unit (CFU-GEMM), granulocyte-macrophage colony-forming unit (CFU-GM), and erythroid burst-forming unit (BFU-E) using a brightfield microscope (Olympus CK40, Olympus). The total number colonies was divided by the number of seeded cells and multiplied by 10^4^ to obtain the number of colonies per 10^4^ HSPC. The percentage of each colony type was also determined, relatively to the total clonogenic potential. Finally, total colony FC was calculated by dividing the total number of colonies at D7 by the total number of colonies at D0.

### Statistical analysis

Data analysis was performed using GraphPad Prism 9 software, except for the transcriptomic data, which was analysed with R. Results from GraphPad were presented as mean ± standard deviation (SD). Shapiro–Wilk tests were carried out to assess data normality for statistical hypothesis testing, followed by one-way analysis of variance (ANOVA) to detect significant differences. For transcriptomic analysis, statistical significance was assessed using p-value adjusted for multiple comparisons by the Benjamini-Hochberg method.

## Results

To elucidate the interactions underlying the benefits of co-culture systems for ex vivo expansion of hematopoietic stem/progenitor cells (HSPC), we investigated the effects of using human platelet lysate (hPL) as a xeno-free alternative to fetal bovine serum (FBS) on mesenchymal stromal cell (MSC) expansion and their subsequent capacity to support the proliferation of HSPC from umbilical cord blood (UCB).

MSC(M) from three independent donors were expanded in vitro using either FBS- or hPL-supplemented media. MSC were cultured under three conditions: (1) continuously in cell isolation medium (**Direct**), (2) adapted from the cell isolation medium to the other medium (Adapted), or (3) re-adapted back to the cell isolation medium (Re-adapted). During MSC expansion, cell morphology, proliferation, immunophenotype, and transcriptomic profiles were evaluated. After four passages, feeder layers (FL) were established for each condition to assess the impact of culture medium supplementation on MSC-mediated support of HSPC. Following a 7-day culture period, proliferation, immunophenotype and clonogenic potential of the expanded HSPC were assessed. Lastly, transcriptomic analysis was conducted to identify differences in gene expression between MSC expanded with FBS-supplemented medium (FBS-MSC) and hPL-supplemented medium (hPL-MSC) aiming to identify key factors influencing the MSC-mediated hematopoietic support of MSC and MSC-HSPC crosstalk (Fig. 1).

### MSC(M) expanded in hPL-supplemented medium display distinct proliferation, morphology, and overall transcriptomic profiles

**Fig. 2:**
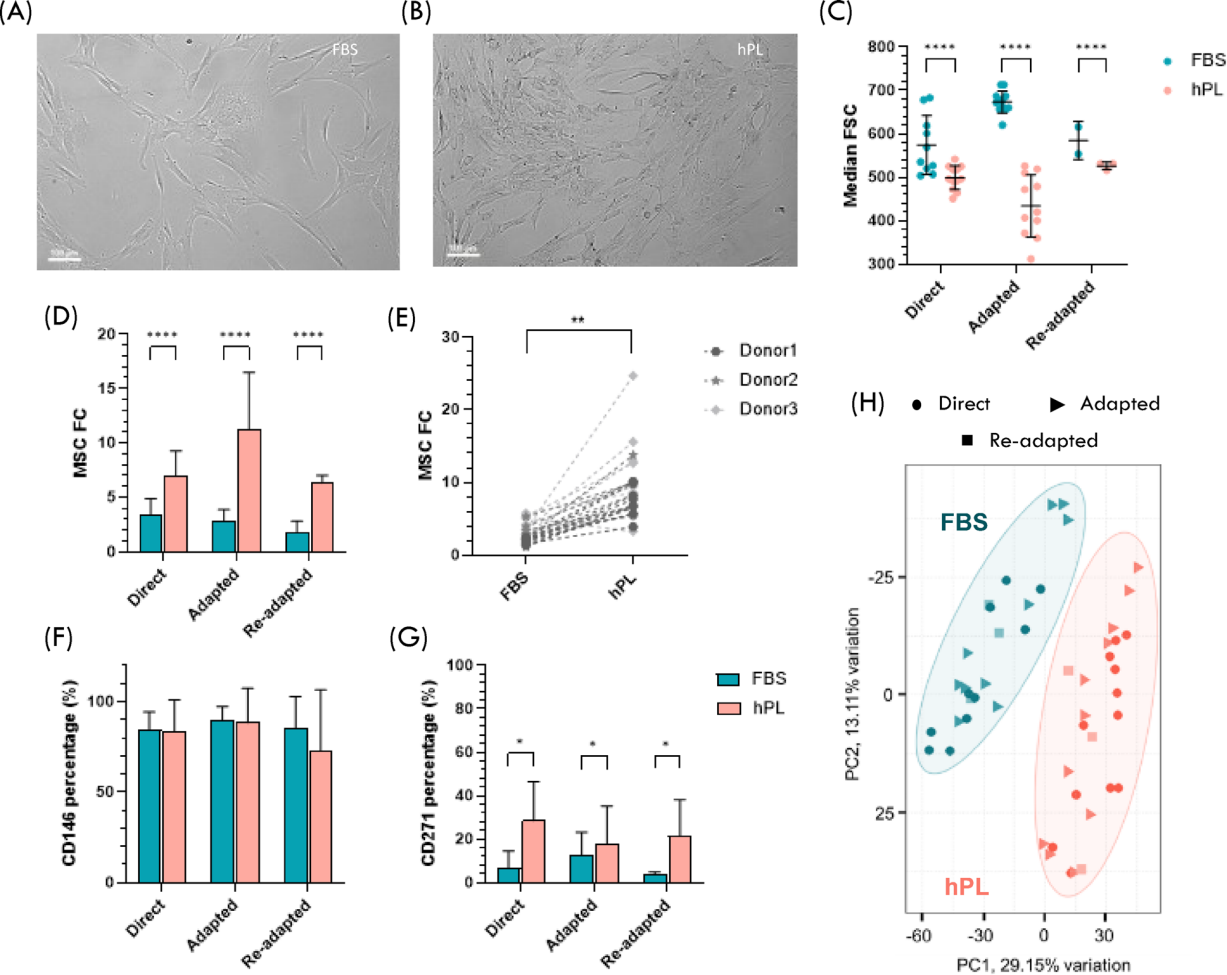
Differential impact of fetal bovine serum (FBS) and human platelet lysate (hPL)-supplementation on mesenchymal stromal cell (MSC) phenotype. Representative brightfield microscopic images of (A) MSC expanded with FBS (FBS-MSC) and (B) expanded with hPL (hPL-MSC) after 4 days in culture. Scale bar represents 100 μm. (C) Median forward scatter (FSC) of FBS-MSC (blue) and hPL-MSC (red) from each adaptation regimen. (D) Fold change (FC) in total cell number for FBS-MSC and hPL-MSC after each passage by adaptation regimen. (E) FC for FBS-MSC and hPL-MSC by donor. Percentage of (F) CD146^+^ MSC and (G) CD271^+^ MSC for FBS-MSC and hPL-MSC by adaptation regimen. (H) Principal component analysis (PCA) of MSC transcriptome with the identification of the FBS-MSC and hPL-MSC cluster. PC1 – Principal component 1; PC2- Principal component 2. Results were obtained for 3 MSC donors (*N* = 3)

hPL-MSC exhibited slightly different morphologic features compared to FBS-MSC. hPL-MSC appeared smaller and more spindle-shaped, whereas FBS-MSC were larger and more flattened (Fig. 2A and B). These differences in size were quantified by the forward scatter (FSC) parameter, which is proportional to cell size, measured during flow cytometry analysis. The same settings were used for both conditions and hPL-MSC were significantly smaller (median FSC: 475 ± 71) compared to FBS-MSC (median FSC: 625 ± 68, p-value < 0.0001) (Fig. 2C). Additionally, hPL-MSC demonstrated a higher proliferation capacity than FBS-MSC, with a consistently higher mean fold change in total cell number per cell passage, regardless of the adaptation regimen (e.g. Direct hPL – 7.0 ± 2.3 vs. Direct FBS – 3.5 ± 1.4) (Fig. 2D) or donor (e.g. hPL-MSC Donor 1–7.2 ± 2.0 vs. FBS-MSC Donor 1–2.0 ± 0.3) (Fig. 2E). Furthermore, hPL-MSC reached higher confluence within the same culture time compared to FBS-MSC (Fig. 2A and B).

 Minimal criteria for MSC characterization defined by the ISCT were successfully confirmed for both FBS-MSC and hPL-MSC, with expression of CD90, CD44, CD73, and CD105 over 95% and expression of CD45, CD34, CD80, CD14, CD11b, CD19, and HLA-DR under 2%. The expression of CD146 (Fig. 2F) and CD271 (Fig. 2E), two markers associated with MSC location within the BM niche and their potency, namely related with hematopoietic cell support [48, 49], was also evaluated. CD146^+^ cell percentage was higher than 70% in all conditions, suggesting that MSC were originally retrieved from the perivascular niche (Fig. 2F). Moreover, CD146 expression was not influenced by the different expansion media employed. CD271 expression levels were consistently lower than those of CD146 (always below 40%) and were significantly increased in hPL-MSC (Fig. 2G).

Principal Component Analysis (PCA) was performed to highlight differences in the transcriptomic machinery of MSC expanded with and without xeno-derived components, while also discerning the impact of several adaptation regimens (Fig. 2H). It was possible to clearly identify two main clusters, regardless of the adaptation regimen or donor, namely one associated with FBS-MSC and another with hPL-MSC. Overall, these results indicate that while hPL is widely used as an FBS alternative for MSC expansion, hPL-MSC and FBS-MSC present phenotypic differences.

### MSC adaptation reveals efficient transcriptional transition between different expansion media

**Fig. 3:**
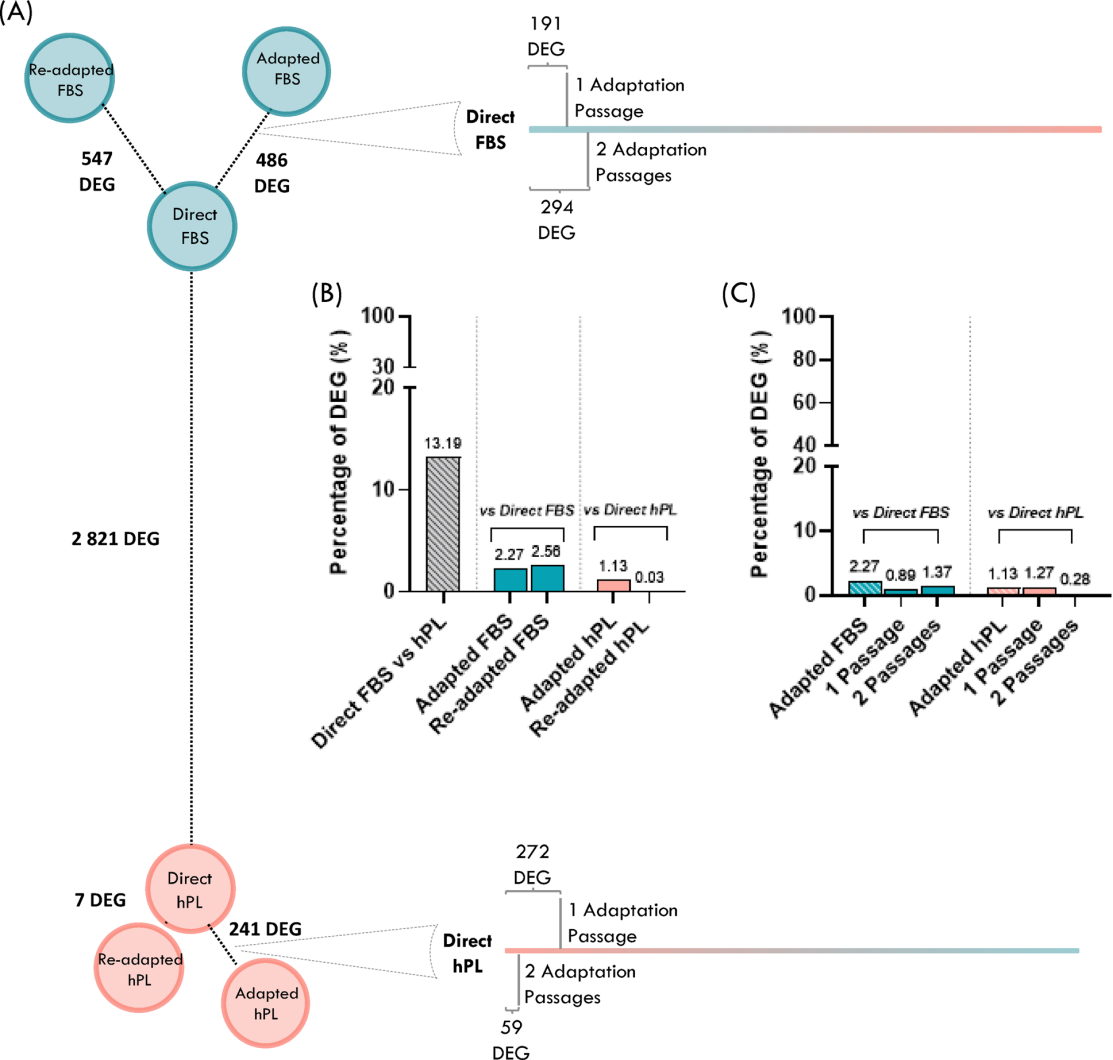
Impact of culture adaptation between human platelet lysate (hPL) and fetal bovine serum (FBS) on the transcriptome of bone-marrow derived mesenchymal stromal cells (MSC(M)). (A) Transcriptional distances (Differentially expressed genes (DEG) number) between MSC derived from each adaptation regimen. DEG were defined as genes with |FC|>2 and padj < 0.05. Distance between conditions is proportional to the number of DEG. Blue circles represent FBS-MSC and red circles hPL-MSC. Percentage of DEG between (B) Direct conditions and Adapted or Re-adapted conditions or (C) Direct conditions and 1 or 2 adaptation passages. Percentages of DEG were obtained by dividing the number of DEG by the number of expressed genes

To evaluate the effectiveness of the adaptation process (i.e. switching media between cell passages) differentially expressed genes (DEG) between MSC expanded under different conditions were computed (Fig. 3A). First, the DEG between Direct FBS and Direct hPL conditions were assessed to validate the differences between FBS-MSC and hPL-MSC. From the 21 393 expressed genes, 2 821 were DEG (13% of the genes) (Fig. 3A and B). Of note, transcriptional differences between hPL-MSC and FBS-MSC were higher than differences between MSC from different donors for each group, confirming the impact of culture media on cell features (Additional File 1, Supplementary Fig. 1).

Then, the number of DEG between Adapted and Direct conditions was determined for both hPL-MSC and FBS-MSC (i.e. Adapted FBS vs. Direct FBS and Adapted hPL vs. Direct hPL). The adaptation process effectively shifted the gene expression profile of Adapted MSC towards that of Direct MSC, indicating that the molecular phenotype induced by the adaptation process resembles that of cells continuously maintained in the same culture medium (Fig. 3A). This effect was particularly observed for hPL adaptations: Direct hPL and Adapted hPL differed by only 1.13% of the genes, whereas Direct FBS and Adapted FBS differed by 2.27% (Fig. 3B).

To understand how the number of adaptation passages influenced the transcriptome, DEG between Direct conditions and MSC that underwent either one or two adaptation passages were evaluated (Fig. 3A and C). For hPL-MSC, the number of DEG decreased after the second adaptation passage (1 adaptation passage: 1.27% DEG, 2 adaptation passages: 0.28% DEG), indicating that two adaptations passages may be beneficial to mimic the behaviour of cells isolated and constantly expanded with hPL. In contrast, the number of DEG increased slightly with the second adaptation passage (1 adaptation passage: 0.89% DEG, 2 adaptation passages: 1.37% DEG) for FBS-MSC (Fig. 3C).

To further understand the plasticity of the transcriptome, MSC were re-adapted back to their original medium. While the FBS re-adaptation process resulted in a comparable number of DEG compared to the initial adaptation (2.56%), hPL re-adaptation produced a transcriptomic profile nearly identical to that of the Direct condition, with only 7 DEG detected between them (0.03%) (Fig. 3A and C).

When more stringent criteria were applied for DEG identification (i.e. |FC| > 4 and padj < 0.01), the number of DEG between Adapted/Re-adapted and Direct conditions decreased more markedly than between the Direct hPL and Direct FBS conditions (Additional File 1, Supplementary Fig. 2). Overall, transcriptomic differences between Adapted/Re-adapted and Direct conditions were minimal - particularly for hPL-MSC - highlighting the effectiveness of the adaptation process to hPL.

### hPL culture reduces total HSPC expansion support but enhances the maintenance of more primitive cell subsets across adaptation regimens

To evaluate the impact of using a xeno-free alternative to expand MSC for their use as feeder layers (FL) in HSPC co-culture expansion, MSC FL were established for all conditions (Direct FBS, Adapted FBS, Re-adapted FBS, Direct hPL, Adapted hPL, and Re-adapted hPL). As a control, HSPC were expanded without FL (No FL) for every expansion run.

**Fig. 4:**
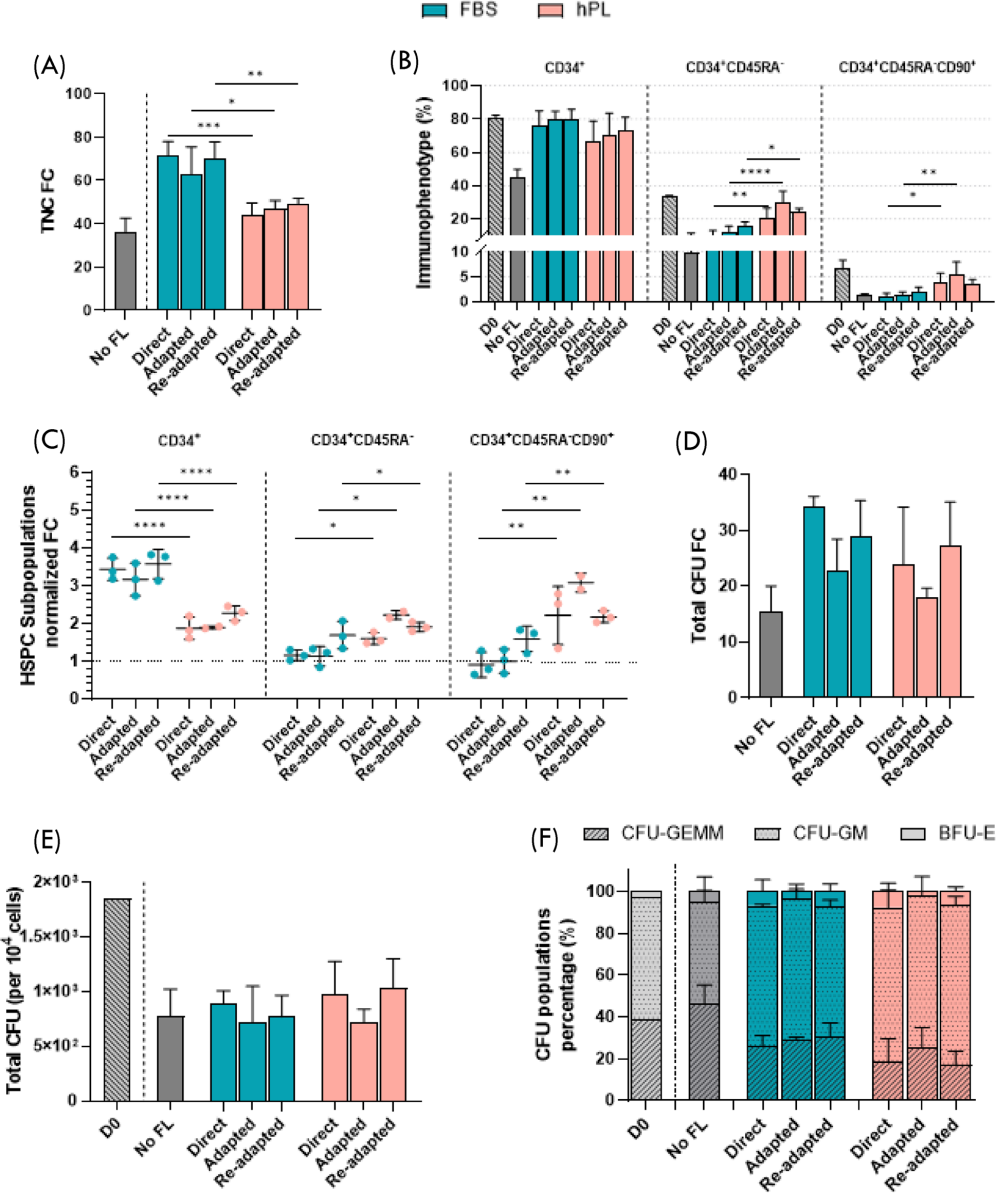
Proliferation, immunophenotype and clonogenic capacity after hematopoietic stem and progenitor cell (HSPC) expansion. (A) Total nucleated cell (TNC) fold change (FC) after expansion of HSPC. Expansion was simultaneously performed without any feeder layer (FL) (control – No FL), with FL of mesenchymal stromal cells (MSC) expanded with fetal bovine serum-supplemented medium (FBS-MSC – blue) and with FL of MSC expanded with human platelet lysate-supplemented medium (hPL-MSC – red) from the three different adaptation regimens. (B) Percentage of CD34^+^, CD34^+^CD45RA^−^, and CD34^+^CD45RA^−^CD90^+^ HSPC before the expansion (D0) and after a 7-day expansion for the different co-culture conditions. (C) FC in CD34^+^, CD34^+^CD45RA^−^, and CD34^+^CD45RA^−^CD90^+^ cells normalized to the No FL condition for each expansion. (D) FC in total number of colony-forming units (CFU) considering TNC obtained from HSPC expansion with the different conditions. (E) Total CFU per 10^4^ cells at D0 and after a 7-day expansion with the different conditions. (E) Percentage of CFU by type at D0 and after a 7-day expansion with the different conditions. Values are presented as mean ± SD. *N* = 8 for ‘No FL’ condition and *N* = 3 for all of the other conditions except for Adapted hPL: *N* = 2

Regardless of the culture medium adaptation regimen, the use of a hPL-MSC FL consistently resulted in a reduced HSPC expansion capacity (Fig. 4A). After a 7-day expansion, total nucleated cell (TNC) number ranged from [(3.8 ± 0.8) to (4.3 ± 0.4)] × 10^6^ in FBS-MSC co-cultures and from [(2.7 ± 0.3) to (2.9 ± 0.2)] × 10^6^ in hPL-MSC co-cultures. Accordingly, FC values in TNC ranged from 62.9 ± 12.6 to 71.8 ± 6.3 for FBS-MSC co-culture systems and from 44.2 ± 5.3 to 48.8 ± 2.9 when hPL-MSC FL were used. In comparison, the No FL control yielded a TNC FC of 35.7 ± 6.9 (Fig. 4A).

The use of hPL-MSC in the co-culture system also modulated several subpopulations of HSPC (Fig. 4B and C) in comparison with FBS-MSC. CD34^+^ cell percentages were higher in FBS-MSC co-cultures [ranging from 75.4 ± 9.5% (Direct FBS) to 79.8 ± 6.0% (Re-adapted FBS)] compared to hPL-MSC co-cultures [ranging from 66.9 ± 11.7% (Direct hPL) to 73.1 ± 7.9% (Re-adapted hPL)] (Fig. 4B). The normalized FC in CD34^+^ cells obtained after expansion on hPL-MSC FL was approximately 1.6 times lower than when using an FBS-MSC FL, with normalization performed against the corresponding No FL control within each experiment (Fig. 4C).

Interestingly, despite the lower overall CD34⁺ expansion, hPL-MSC FL promoted significantly higher enrichment of more primitive HSPC subsets. Both the percentage and normalized FC of CD34^+^CD45RA^−^ (p-value < 0.0005 and p-value < 0.005, respectively) and CD34^+^CD45RA^−^CD90^+^ (p-value < 0.005 and p-value < 0.0005, respectively) populations were increased in hPL-MSC co- cultures relative to FBS-MSC (Fig. 4B and C).

Finally, clonogenic assays indicated no significant differences in progenitor functionality between conditions. Total number of colony forming units (CFU) per 10^4^ cells (Fig. 4E), percentage of CFU types (Fig. 4F), and total CFU FC (Fig. 4D) were comparable across all conditions, not demonstrating a clear correlation with the culture medium used to prepare MSC FL.

### Transcriptional and signaling alterations in hPL-MSC highlight potential implications for MSC metabolism and for MSC-HSPC crosstalk

**Fig. 5:**
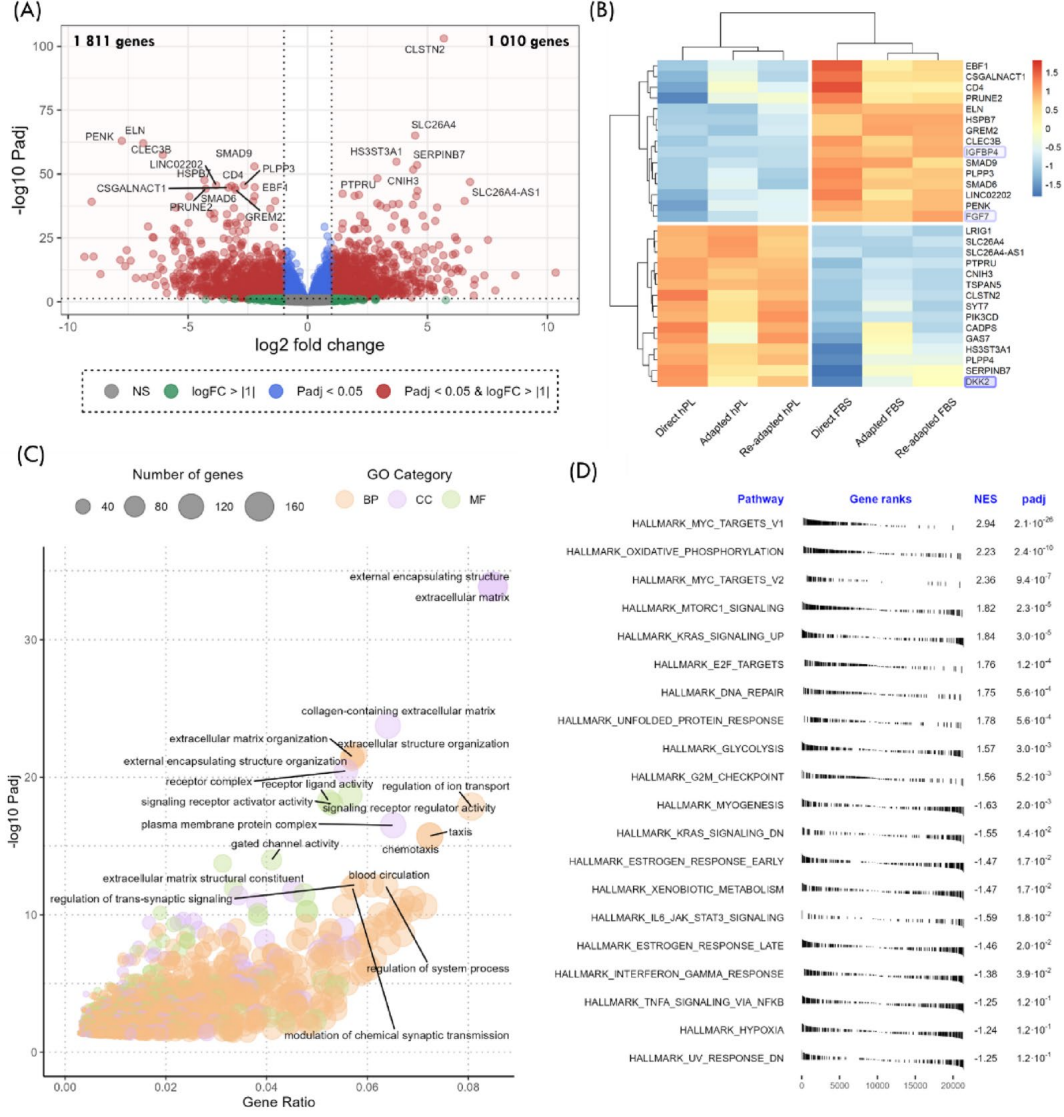
Transcriptomic differences between mesenchymal stromal cells (MSC) isolated and expanded with human platelet lysate (Direct hPL) and MSC isolated and expanded with fetal bovine serum (Direct FBS) (*n* = 12). (A) Volcano Plot with all expressed genes. Differentially expressed genes (DEG) (|FC| >2, padj < 0.05) are identified by red dots and the top 20 DEG are labeled. (B) Heat plot normalized for each gene (i.e. by row) of the top 15 downregulated genes and top 15 upregulated genes ordered by their Walt statistic. (C) Bubble plot with gene ontology results. Top 20 ontologies are labeled. (D) Ranked results of gene set enrichment analysis for the hallmark pathways. FC – fold change, padj – p-value adjusted for multiple comparisons, GO – gene ontology, NES – normalized enrichment score

To understand the main transcriptomic differences between hPL-MSC and FBS-MSC, data from the Direct conditions for both media were analyzed. Firstly, DEG (|FC| >2 and padj < 0.05) were determined, originating a list of 2 821 genes, from which 1 811 were downregulated and 1 010 upregulated in hPL-MSC (Fig. 5A, Additional File 3). Of note, by analyzing the top 30 DEG ordered by padj (Fig. 5B), 3 genes encoding HSPC regulation factors were identified – IGFBP4, FGF7, and DKK2.

To identify key biological processes altered in hPL-MSC, gene ontology (GO) analysis (Fig. 5C) and gene set enrichment analysis (GSEA) based on Hallmark pathways (Fig. 5D) were performed. Most affected ontologies were related to extracellular matrix organization/composition but also related to taxis and cell signaling/receptor activity. GSEA of the hallmark pathways concluded that most of the top gene sets affected were related to cell metabolism.

### MSC function-based DEG analysis reveals cell adhesion, signaling, and TGF-β pathways underlying impaired HSPC expansion support

**Fig. 6:**
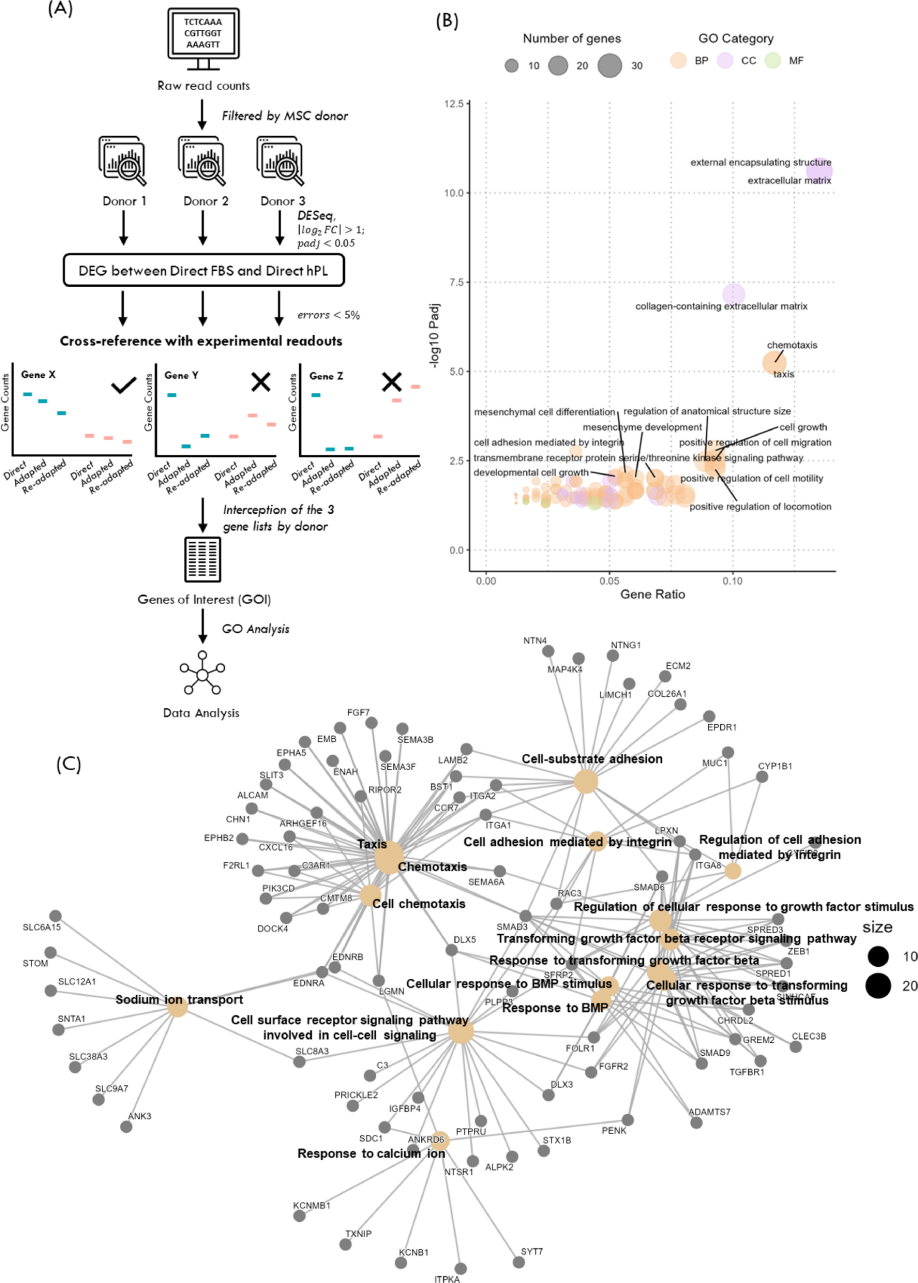
Top-down approach. (A) Schematic of the transcriptomic workflow to reach genes of interest (GOI). Initially, reads were filtered by donor and differentially expressed genes (DEG) were determined. Genes from the initial DEG list that had a coherent expression over the different adaptation regimens for all donors, were labelled as GOI. (B) Bubble plot with gene ontology (GO) results for GOI. Top 20 ontologies are labeled. (C) CNET plot depicting the interactions between genes from the different ontologies related with cell-cell signaling

To narrow the pool of DEG in search of genes responsible for the hematopoietic support capacity of MSC, we took into consideration the experimental readouts of the various HSPC expansion co-cultures. Since co-cultures established with FL from hPL-MSC (Direct, Adapted and Re-adapted) consistently showed reduced TNC FC and CD34^+^ expansion, we focused on genes whose expression patterns matched this experimental phenotype. For each donor, DEG identified between Direct hPL and Direct FBS, were further filtered based on their expression in the Adapted and Re-adapted conditions. Specifically, to pass through this filter, a gene upregulated in Direct FBS-MSC had also to be expressed at higher levels in both Adapted and Re-adapted FBS-MSC compared to all hPL-MSC conditions; the same logic was applied for downregulated genes. The final list of genes of interest (GOI) was obtained by intercepting cross-referenced genes between the three donors (Fig. 6A). In total, 322 GOI were identified (180 downregulated and 142 upregulated in hPL-MSC).

GO analysis was performed to understand the main properties of MSC affected by GOI (Fig. 6B). Once more, extracellular matrix structure/composition was identified as one of the affected categories, but the number of GO terms related with cell signaling, mobility, or adhesion increased in comparison to using all DEG. Of note, mesenchymal cell differentiation and development of mesenchyme were also within the top 15 affected ontologies. Focusing on cell-cell communication, interaction between different GO terms was also assessed (Fig. 6C).

Signaling pathways as TGF-β and cell adhesion regulation by integrins shared multiple genes among them, indicating that all of these should have similar activation/inhibition status between hPL-MSC and FBS-MSC (Fig. 6C). Thus, the alterations observed in these pathways are probable causes for the decrease in the capacity of hPL-MSC to support the overall expansion of HSPC in the conditions of our study.

### hPL-MSC show altered expression of HSPC regulatory genes linked to PI3K-Akt, TGF-β, and Wnt pathways

**Fig. 7:**
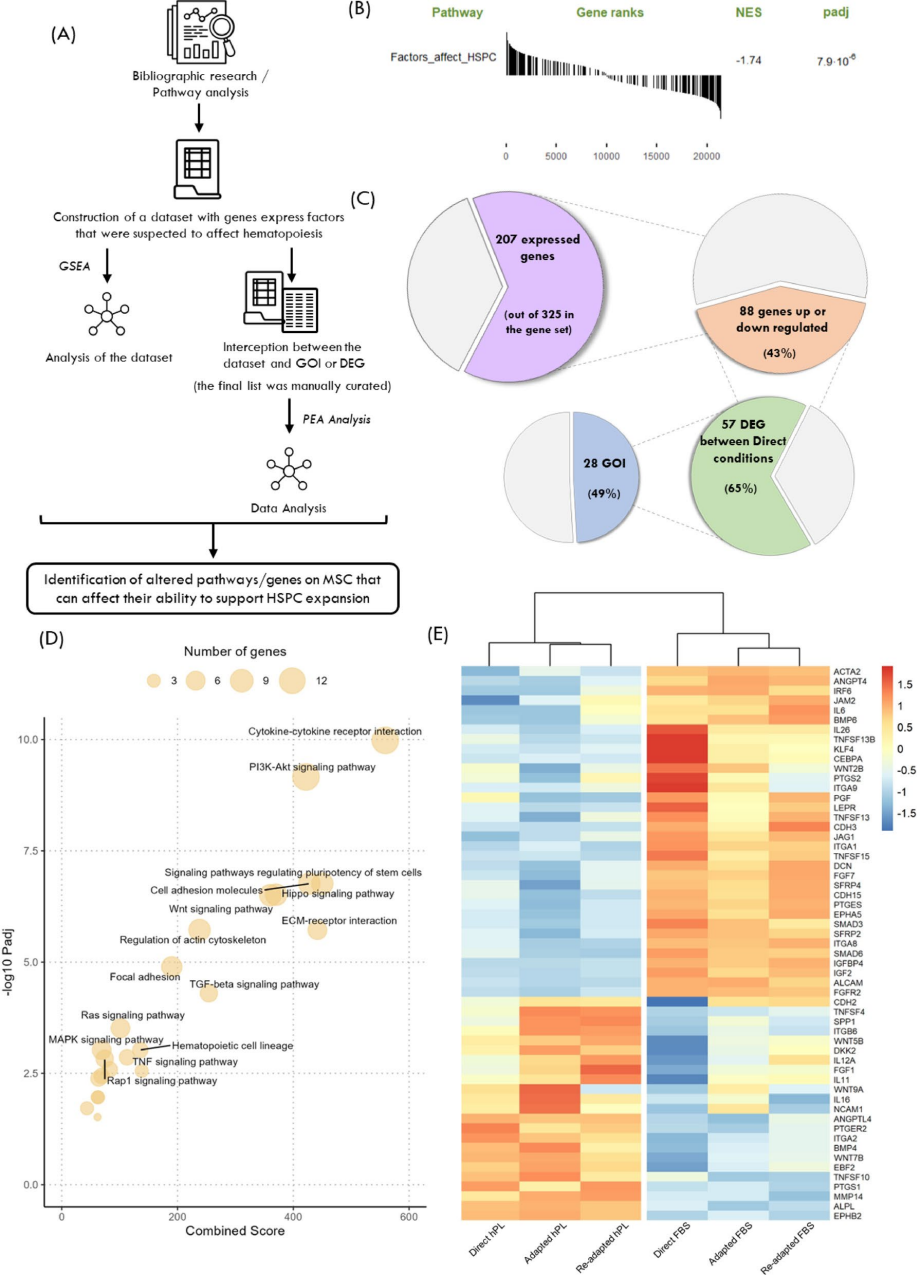
Bottom-up approach. (A) Scheme of the transcriptomic analysis. A dataset of genes encoding paracrine factors involved in hematopoietic stem/progenitor cells (HSPC) regulation was build (HSPC regulation gene set). The expression of these genes was analyzed across conditions and the list of genes of interest (GOI) and differentially expressed genes (DEG) were intercepted with this dataset. Pathway enrichment analysis (PEA) was performed on the final list of genes. (B) Gene set enrichment analysis (GSEA) of the HSPC regulation gene set. (C) Expression profile of the genes on the HSPC regulation gene set. (D) Bubble plot with PEA results for the final list of genes. KEGG pathways were used and the top 20 pathways labeled. (E) Heat plot of the list of GOI/DEG present in the dataset normalized by gene (row)

After reaching a phenotype-confirmed set of genes (i.e. GOI), we decided to further investigate the relationship between DEG and/or GOI and the altered capacity of hPL-MSC to support HSPC during ex vivo expansion (Fig. 7A). To do so, a gene set with 325 genes that encode factors that have been previously described to be involved in the regulation of HSPC was assembled, which from now on will be referenced as “HSPC regulation gene set” (Additional File 2).

Analysis of the HSPC regulation gene set was performed by GSEA obtaining a normalized enrichment score (NES) of −1.74, indicating that FBS-MSC are enriched for these genes in comparison to hPL-MSC (Fig. 7B).

When looking at the genes in the HSPC regulation gene set, it was possible to see that 64% of them were expressed by MSC. From those, more than 40% presented differences in the expression between hPL-MSC and FBS-MSC, despite not all being differentially expressed, 65% were classified as DEG (|FC| > 2, padj < 0.05). Around half of the identified DEG were included in the GOI, confirming significant differences in the expression of genes encoding factors associated with the regulation of HSPC (Fig. 7C). Expression of DEG or GOI present in the HSPC regulation gene set was assessed. Most of them presented similar expression between the conditions of the same culture medium, with Direct FBS typically having the highest expression for genes upregulated and the lowest expression for genes downregulated in FBS-MSC (Fig. 7E).

Lastly, DEG or GOI present in the HSPC regulation gene set were used to perform pathway enrichment analysis (PEA) to further understand which pathways were altered in hPL-MSC, leading to differential expression of genes involved in MSC-HSPC crosstalk (Fig. 7D). Pi3K-Akt signalling pathway stood as the one more significantly affected, but several others, such as TGF-β or Wnt signalling pathway, also appeared to be relevant. Interestingly, cytokine-cytokine receptor interactions and ECM-receptor interactions were also enriched, supporting the influence of ECM components and cell-cell communication mechanisms proposed before for MSC-HSPC interactions.

These results suggest that hPL-MSC support to HSPC is altered by changes across key pathways involved in both paracrine and contact-dependent mechanisms.

### Distinct MSC-derived signals from hPL and FBS cultures influence key regulatory pathways in HSPC

Factors potentially affecting HSPC proliferation and their associated pathways were first identified through unbiased analysis and then validated by cross-checking the DEG and GOI lists with a curated gene set of hematopoietic regulatory factors known to be expressed by supportive cells.

**Fig. 8:**
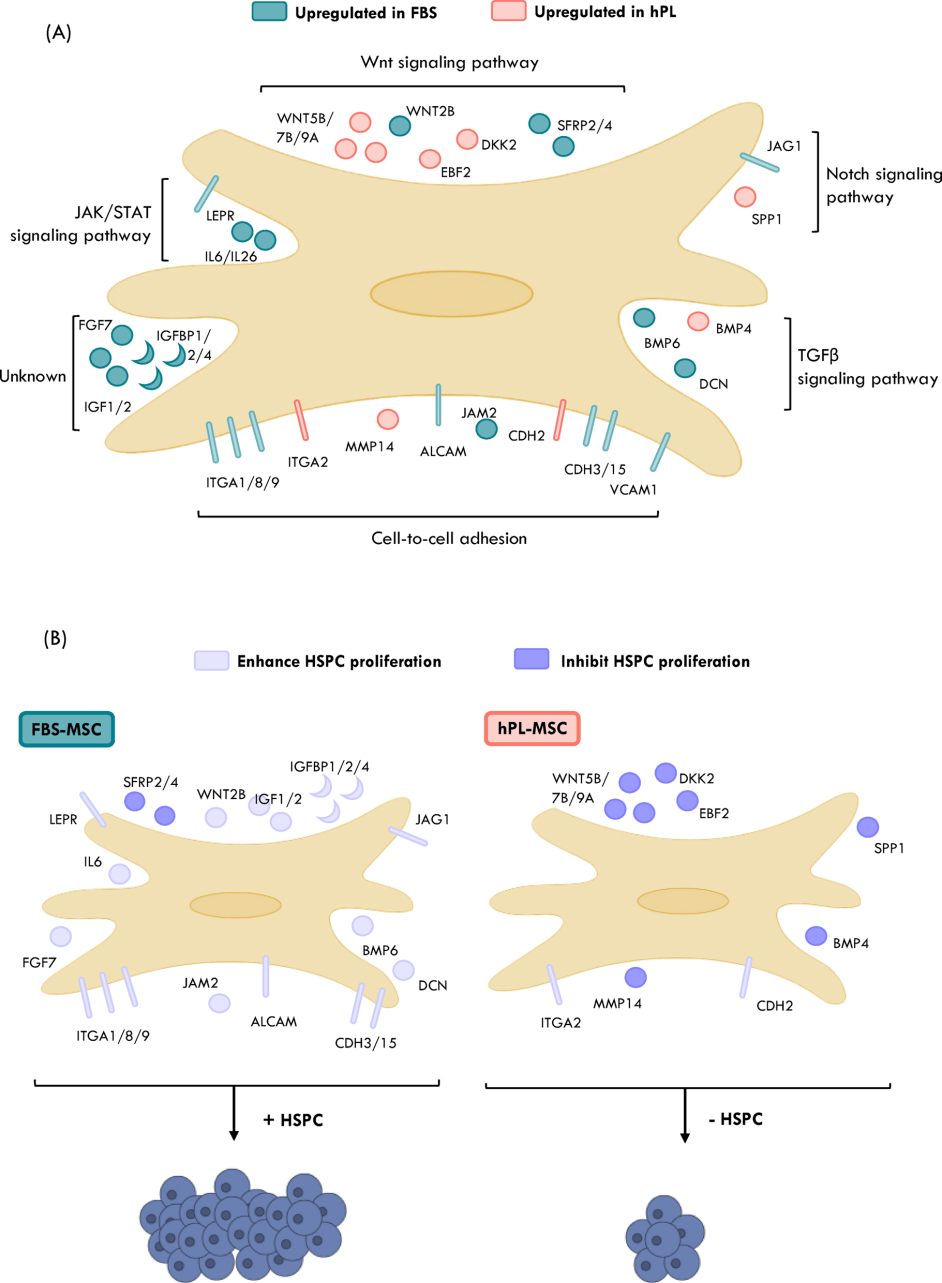
Proposed signaling pathways that are responsible for supporting hematopoietic stem and progenitor cell (HSPC) expansion through mesenchymal stromal cell (MSC) co-culture. (A) Schematic with genes encoding factors responsible for HSPC regulation and their pathways; blue - genes upregulated in MSC expanded with fetal bovine serum supplemented medium (FBS-MSC), red - genes upregulated in MSC expanded with human platelet lysate (hPL-MSC). (B) Genes upregulated in more supportive FBS-MSC (left) and less supportive hPL-MSC (right). Light purple: genes that encode factors known to enhance HSPC proliferation. Dark purple: genes encoding factors known to inhibit HSPC proliferation

Our findings suggest that when hPL-MSC are used as FL, key signaling pathways such as Notch, TGF-β, Wnt, JAK/STAT, as well as cell-cell adhesion mechanisms, could be affected in HSPC, leading to their lower proliferation (Fig. 8A).

FBS-MSC mostly overexpressed genes encoding factors previously reported to enhance HSPC proliferation, such as *JAG1*, *BMP6*, *DCN*, *CDH3*, *CDH15*, *ALCAM*, *JAM2*, *ITGA1*, *ITGA8*, *ITGA9*, *IL6*, *LEPR*, *WNT2B*, *FGF7*, *IGF1*, *IGF2*, *IGFBP1*, *IGFBP2*, and *IGFBP4* (Additional File 1, Supplementary Fig. 3). Notably, two genes encoding factors with reported inhibitory effects on HSPC proliferation were also upregulated in FBS-MSC (*SFRP2* and *SFRP4*).

On the other hand, hPL-MSC showed higher expression of genes encoding factors known to suppress HSPC proliferation, including *SPP1*, *MMP14*, *BMP4*, *DKK2*, *EBF2*, *WNT5B*, *WNT7B*, and *WNT9A* (Additional File 1, Supplementary Fig. 4). There were only two genes encoding factors reported to enhance HSPC proliferation overexpressed in hPL-MSC (i.e. *ITGA2* and *CDH2*).

Altogether, these results reveal a shift in the expression of genes involved in paracrine signaling and direct cell contact, suggesting specific molecular pathways underlying the altered hematopoietic support capacity of hPL-MSC.

## Discussion

Overcoming the challenges of donor matching and the increasing demand for suitable HSPC sources are essential to improve HCT outcomes. HSPC(CB) offer distinct advantages over adult sources, including lower rates of graft-versus-host disease and less stringent HLA matching requirements [1]. However, due to their limited collection volumes, they often require ex vivo expansion for use in adult patients [3, 4]. In this context, the FDA has recently approved Omisirge, the first cell therapy product for blood cancers based on UCB [20]. This approval strengthens the application of ex vivo expanded HSPC in clinical transplantation. Among the various systems established for HSPC ex vivo expansion, MSC-HSPC co-cultures stand out for their ability to recapitulate key cellular interactions of the BM niche. These co-culture systems not only support HSPC proliferation and maintenance but also provide a valuable platform to study MSC-HSPC crosstalk, which is important for the development of improved HSPC ex vivo expansion platforms.

In line with cGMP, the use of xeno-free supplements, such as hPL, is increasingly being adopted as a substitute for FBS in MSC manufacturing [50]. However, our group previously reported that MSC isolated in FBS-supplemented medium and then expanded in hPL-supplemented medium (Adapted hPL-MSC) exhibited a reduced ability to support ex vivo expansion of HSPC [40]. In the current study, we conducted a comprehensive comparison between hPL-MSC and FBS-MSC and investigated the effects of different adaptation regimens, integrating functional co-culture assays with transcriptomic analysis.

We first confirmed that hPL-MSC consistently led to lower TNC number and lower CD34^+^ cells percentages after hematopoietic cell expansion, regardless of the adaptation regimen. Interestingly, despite the overall lower number of HSPC obtained, immunophenotypic analysis indicated that hPL-MSC promoted a higher enrichment of more primitive cell subsets. When analyzing clonogenic capacity, hPL-MSC led to a lower total number of CFU, reflecting the reduced cell yield. However, normalized CFU for the same number of HSPC were slightly higher (although not statistically significant), suggesting that individual cells retain comparable or potentially enhanced clonogenic potential. Importantly, HSPC were never exposed to either hPL- or FBS-supplemented medium; all cultures were performed in the same serum-free medium (StemSpan SFEM II), ensuring that the differences in hematopoietic cell expansion outcomes can be attributed to the feeder layer phenotype rather than intrinsic loss of HSPC functionality.

Transcriptomic analysis revealed that a single cell passage following direct media change was sufficient to induce major gene expression shifts, which were rapidly reversed upon re-adaptation in the subsequent cell passage. Most adaptation protocols involve multiple passages, either by gradually decreasing serum concentration before transitioning to a serum-free medium or by progressively adapting cells between media [35]. In this study, adaptations were performed by directly replacing the entire medium volume with the target medium. This provides practical advantages for the manufacturing of cell-based products. For example, MSC isolated using different media could be standardized by adapting them to a common culture condition, enabling the use of xeno-free or chemically defined formulations that may be unsuitable for initial cell isolation. From a cGMP perspective, reducing the number of adaptation passages minimizes process complexity, time and variability, thereby increasing reproducibility and scalability of MSC manufacturing. Furthermore, since a single passage was sufficient to induce transcriptomic remodeling, production costs could be reduced without compromising cell functionality.

Consistent with our findings, previous reports have shown that even short exposure (i.e. 2 days) to an alternative culture medium can induce differential gene expression, with particularly pronounced differences between FBS-MSC vs. hPL-MSC [51]. Moreover, these studies show that MSC undergo differences in proliferation, cytoskeletal organization, and focal adhesion dynamics after short term exposure, and that these alterations are reversible upon switching back to the original medium [51]. It should be noticed that researchers often change the culture medium upon cell isolation or during expansion without considering the potential impact on cell identity and function. Such changes can lead to cellular stress or unintended phenotypic shifts.

Several studies have previously demonstrated that MSC expanded in different culture media exhibit distinct phenotypic and functional properties [52, 53]. For instance, hPL-MSC are typically more proliferative and morphologically distinct compared to FBS-MSC, whereas altered differentiation capacity, impaired immunomodulatory potential, and impaired angiogenic properties have also been reported [37–39]. These functional differences correlate with the distinct transcriptomic profiles we identified between hPL-MSC and FBS-MSC. Only two studies have previously compared the transcriptomes of hPL-MSC and FBS-MSC. One reported minimal differences between these MSC populations, possibly underestimated due to high variability in in-house produced hPL derived from a limited donor pool [51]. In contrast, the other study, consistent with our findings, identified substantial differences between hPL-MSC and FBS-MSC, particularly in ECM-related pathways [38]. Our GO analysis confirmed this by identifying ECM organization and composition as highly enriched categories. Supporting these findings, adipose tissue and Wharton’s jelly derived hPL-MSC or FBS-MSC exhibited differences in adhesion stability as well as ECM composition and deposition [54, 55].

Despite extensive efforts to elucidate HSPC-niche interactions within the BM, namely the role of supportive MSC, much remains unknown. Nonetheless, several mechanisms have been established as essential for MSC-HSPC interactions, including cell-cell contact mediated by different adhesion molecules [25, 26, 56, 57], as well as the secretion of key cytokines, chemokines and growth factors [58, 59]. To further investigate the mechanisms underlying the MSC-mediated hematopoietic support, we focused on a subset of DEG that were validated by our experimental data (i.e. GOI). Beyond ECM composition and organization, we identified significant alterations in gene ontologies related to integrin-mediated cell adhesion mechanisms, cell-cell communication, and the TGF-β signaling pathway, which might be important for HSPC regulation.

To further explore whether the identified genes were critical for HSPC regulation, we generated a novel gene set comprising 325 genes encoding signaling factors likely implicated in HSPC regulation (e.g., proliferation, differentiation, homing) based on literature curation [60–69]. Our analysis revealed significant differences in the expression of these genes between hPL-MSC and FBS-MSC, consistent with the experimental findings described above. Importantly, many supportive genes were downregulated in hPL-MSC while inhibitory genes were upregulated [25, 26, 56–59]. Of note, *IGFBP4* and *FGF7* (supportive) were among the top 15 DEG upregulated in FBS-MSC, whereas DKK2 (inhibitory) was among the top 15 DEG upregulated in hPL-MSC. Pathway enrichment analysis further highlighted dysregulation of the Pi3K-Akt, TGF-β and Wnt signaling pathways, as well as cytokine-cytokine and ECM-receptor interactions. These alterations suggest that MSC culture conditions directly affect key signaling axes critical for HSPC maintenance and expansion. Overall, rather than identifying a single dominant mechanism, we found differences in the expression of cues regulating multiple pathways involved in HSPC proliferation and differentiation, including Notch [70, 71], TGF-β [72, 73], Wnt [74–77], and JAK/STAT [78, 79] signaling pathways, as well as cell-cell adhesion mechanisms [80–84].

Although these changes have been assessed at the transcript level, functional validation at the protein level would provide additional mechanistic insights and represents an important direction for future work. Importantly, some of the genes identified have been functionally validated in previous studies: JAM2-deficient MSC showed impaired hematopoietic regulation in vivo (murine model) [85], while JAG1 overexpression in MSC enhanced CD34^+^ cell proliferation in vitro (human in vitro model) [86], supporting the biological relevance of our transcriptomic findings. Future studies should further validate the proposed mechanisms through targeted in vitro assays, including gene knockdown/overexpression of these and other candidates.

Our study not only reinforces the biological relevance of transcriptomic profiling in assessing MSC function but also provides important translational insights into MSC-HSPC interactions within the complex BM niche. In addition, we identified limitations associated with xeno-free alternatives for HSPC expansion. As compliance with current cGMP becomes increasingly important, pinpointing problematic signaling pathways could facilitate the development of improved xeno-free formulations. By elucidating key gene expression changes induced by MSC culture conditions, our findings support the design of novel xeno-free media with enhanced hematopoietic supportive properties. Although beyond the scope of the current study, our transcriptomic dataset provides a shortlist of candidate factors that could be tested in future chemically defined media formulations, potentially enabling the recapitulation of FBS-MSC support without xenogeneic supplements. Moreover, a deeper understanding of MSC-HSPC interactions may enable the development of synthetic signaling approaches, in which key supportive signals are mimicked without requiring live feeder layers, improving HSPC ex vivo expansion for clinical use. Furthermore, the ability to tune HSPC phenotypic changes by priming MSC feeder layer could allow precise control over the composition of HSPC products, enabling optimization for specific therapeutic indications. In summary, while FBS-MSC provided superior biological support for HSPC expansion, hPL-MSC remain a promising cGMP-compatible alternative. Choosing between the two media involves balancing biological performance with regulatory and manufacturing advantages, and our data highlight specific molecular pathways that could be targeted to narrow this gap.

Overall, this study provides valuable insights into MSC-HSPC interactions and offers translational knowledge for optimizing MSC manufacturing using hPL and FBS supplementation. Continued research in this field will enhance our understanding of the intricate crosstalk between HSPC and MSC, ultimately advancing strategies for HSPC expansion and their applications in hematopoietic cell transplantation and regenerative medicine settings.

## Conclusion

The development and optimization of efficient HSPC(CB) expansion systems are essential to overcome the current challenges in hematopoietic cell transplantation. Although UCB represents a valuable alternative to adult HSPC sources, mitigating issues such as limited donor availability and poor HLA matching, it still requires ex vivo expansion to achieve clinically relevant cell number for adult patients. Given that MSC-HSPC co-culture remains one of the most extensively studied expansion strategies, with clinical trials using FBS-MSC showing promising results and improved engraftment, gaining a deeper understanding of their interaction dynamics is crucial.

In this study, we demonstrated that the choice of culture supplement (FBS vs. hPL) induces significant and reversible functional, phenotypic, and transcriptomic changes in MSC that should not be underestimated. The experimentally observed differences in the ability of FBS-MSC and hPL-MSC to support HSPC expansion ex vivo were corroborated at the transcriptomic level, with corresponding differences in the expression of key regulatory genes involved in cell adhesion, cytokine signaling, and niche-related pathways.

Based on these findings, we developed a model that increases our understanding of MSC-mediated HSPC support, providing a rational basis for optimizing HSPC expansion systems. Nevertheless, further studies are essential to validate the proposed mechanisms and functionally characterize the identified gene candidates to fully elucidate their roles in HSPC regulation.

Overall, this work advances the knowledge of MSC-HSPC interactions, highlights critical considerations for MSC manufacturing and ultimately contributes to the refinement of HSPC expansion protocols, facilitating their effective translation into clinical applications.

## Supplementary Information

Supplementary Material 1. Supplementary figures and tables. Composed of supplementary Tables 1 and supplementary Figs. 1–4.

Supplementary Material 2. MSC_genes_affect_HSPC.xlsx – Geneset of genes encoding factors previously reported to impact the expansion of HSPC.

Supplementary Material 3. DEG_DirectFBS_vs_DirecthPL.xlsx – Differential Expressed Genes between Direct FBS-MSC and Direct hPL-MSC. Includes gene name, baseMean (mean of normalized counts of all samples, normalizing for sequencing depth), log2FoldChange (base 2 logarithm of the fold change between Direct FBS and Direct hPL), lfcSE (standard error estimate for the log2 fold change estimate), stat (Wald statistic), pvalue, and padj (p-value adjusted for multiple testing).

## Data Availability

All data are available on request. All additional files are included in the manuscript. RNA_Seq data is available in the GEO repository under the accession number GSE294580.

## Abbreviations

UCB: Umbilical cord blood
HSPC: Hematopoietic stem and progenitor cells
MSC: Mesenchymal stromal cells
FBS: Fetal Bovine Serum
hPL: human Platelet Lysate
FBS-MSC: MSC expanded with FBS-supplemented medium
hPL-MSC: MSC expanded with hPL-supplemented medium
HSPC(CB): UCB-derived HSPC
BM: Bone Marrow
MSC(M): BM derived MSC
MNC: Mononuclear Cells
MNC(CB): UCB-derived MNC
HCT: Hematopoietic Cell Transplantation
HLA: Human Leukocyte Antigen
ECM: Extracellular Matrix
FL: Feeder Layers
cGMP: current Good Manufacturing Practices
A.A.: Antibiotic-Antimycotic
DMEM: Dulbecco’s Modified Eagle’s Medium
DMEM/FBS: DMEM supplemented with 10% FBS and 1% A.A
DMEM/hPL: DMEM supplemented with 5% hPL and 1% A.A
L/D: LIVE/DEAD Fixable Far Red Dead Cell Stain
DMSO: Dimethyl Sulfoxide
MACS: Magnetic Activated Cell Sorting
CFU: Colony-Forming Unit
CFU-GEMM: multilineage CFU
CFU-GM: Granulocyte-Macrophage CFU
BFU-E: Erythroid Burst-Forming Unit
SCF: Stem Cell Factor
Flt-3 L: Fms-like Tyrosine Kinase 3 Ligand
TPO: Thrombopoietin
bFGF: basic Fibroblast Growth Factor
TNC: Total Nucleated Cells
FSC: Forward Scatter
DEG: Differential Expressed Genes
PCA: Principal Component Analysis
FC: Fold Change
padj: p-value for multiple testing
GSEA: Gene Set Enrichment Analysis
GOI: Genes of Interest
GO: Gene Ontology
PEA: Pathway Enrichment Analysis
NES: Normalized Enrichment Score
SD: Standard Deviation
ANOVA: Analysis of Variance
FDA: U.S. Food and Drug Administration
ISCT: International Society for Cell and Gene Therapy

## References

[1] Gabelli M, Veys P, Chiesa R. Current status of umbilical cord blood transplantation in children. Br J Haematol. 2020;190(5):650–83. 10.1111/bjh.16107. 31410846 10.1111/bjh.16107

[2] Gluckman E, Rocha V. Cord blood transplantation: state of the art. Haematologica. 2009;94(4):451. 19336748 10.3324/haematol.2009.005694 PMC2663606

[3] Kiernan J, Damien P, Monaghan M, Shorr R, McIntyre L, Fergusson D, et al. Clinical studies of ex vivo expansion to accelerate engraftment after umbilical cord blood transplantation: a systematic review. Transfus Med Rev. 2017;31(3):173–82. 28087163 10.1016/j.tmrv.2016.12.004

[4] Delaney C, Ratajczak MZ, Laughlin MJ. Strategies to enhance umbilical cord blood stem cell engraftment in adult patients. Expert Rev Hematol. 2010;3(3):273. 20835351 10.1586/ehm.10.24 PMC2935587

[5] Branco A, Rayabaram J, Miranda CC, Fernandes-Platzgummer A, Fernandes TG, Sajja S, et al. Advances in *ex vivo* expansion of hematopoietic stem and progenitor cells for clinical applications. Front Bioeng Biotechnol. 2024;12:1380950. 38846805 10.3389/fbioe.2024.1380950 PMC11153805

[6] Sotnezova EV, Andreeva ER, Grigoriev AI, Buravkova LB. Ex vivo expansion of hematopoietic stem and progenitor cells from umbilical cord blood. Acta Nat. 2016;8(3):6. PMC5081707 27795840

[7] Costa MHG, de Soure AM, Cabral JMS, Ferreira FC, da Silva CL. Hematopoietic Niche–exploring biomimetic cues to improve the functionality of hematopoietic stem/progenitor cells. Biotechnol J. 2018;13(2):1700088. 10.1002/biot.201700088. 10.1002/biot.201700088 29178199

[8] Branco A, Bucar S, Moura-Sampaio J, Lilaia C, Cabral JMS, Fernandes-Platzgummer A, et al. Tailored cytokine optimization for *ex vivo* culture platforms targeting the expansion of human hematopoietic Stem/Progenitor cells. Front Bioeng Biotechnol. 2020;8:1154. 10.3389/fbioe.2020.573282 PMC7729524 33330414

[9] Andrade PZ, Dos Santos F, Almeida-Porada G, Lobato Da Silva C, Joaquim JM. Systematic delineation of optimal cytokine concentrations to expand hematopoietic stem/progenitor cells in co-culture with mesenchymal stem cells. Mol Biosyst. 2010;6(7):1207–15. 20424784 10.1039/b922637k

[10] Dahlberg A, Delaney C, Bernstein ID. Ex vivo expansion of human hematopoietic stem and progenitor cells. Blood. 2011;117(23):6083. 21436068 10.1182/blood-2011-01-283606 PMC3122936

[11] Lampreia FP, Carmelo JG, Anjos-Afonso F. Notch signaling in the regulation of hematopoietic stem cell. Curr Stem Cell Rep. 2017;3(3):202. 28845387 10.1007/s40778-017-0090-8 PMC5548842

[12] Delaney C, Varnum-Finney B, Aoyama K, Brashem-Stein C, Bernstein ID. Dose-dependent effects of the Notch ligand Delta1 on ex vivo differentiation and in vivo marrow repopulating ability of cord blood cells. Blood. 2005;106(8):2693–9. 15976178 10.1182/blood-2005-03-1131 PMC1366491

[13] Ventura Ferreira MS, Jahnen-Dechent W, Labude N, Bovi M, Hieronymus T, Zenke M, et al. Cord blood-hematopoietic stem cell expansion in 3D fibrin scaffolds with stromal support. Biomaterials. 2012;33(29):6987–97. 22800538 10.1016/j.biomaterials.2012.06.029

[14] Chua KN, Chai C, Lee PC, Tang YN, Ramakrishna S, Leong KW, et al. Surface-aminated electrospun nanofibers enhance adhesion and expansion of human umbilical cord blood hematopoietic stem/progenitor cells. Biomaterials. 2006;27(36):6043–51. 16854459 10.1016/j.biomaterials.2006.06.017

[15] Wasnik S, Kantipudi S, Kirkland MA, Pande G. Enhanced ex vivo expansion of human hematopoietic progenitors on native and spin coated acellular matrices prepared from bone marrow stromal cells. Stem Cells Int. 2016. 10.1155/2016/7231567. 26981135 10.1155/2016/7231567 PMC4769778

[16] Li J, Wang X, Ding J, Zhu Y, Min W, Kuang W, et al. Development and clinical advancement of small molecules for ex vivo expansion of hematopoietic stem cell. Acta Pharm Sin B. 2022;12(6):2808–31. 35755294 10.1016/j.apsb.2021.12.006 PMC9214065

[17] Wagner JE, Brunstein CG, Boitano AE, Defor TE, McKenna D, Sumstad D, et al. Phase I/II Trial of stemregenin-1 expanded umbilical cord blood hematopoietic stem cells supports testing as a stand-alone graft. Cell Stem Cell. 2016;18(1):144–55. 26669897 10.1016/j.stem.2015.10.004 PMC4881386

[18] Cohen S, Roy J, Lachance S, Delisle JS, Marinier A, Busque L, et al. Hematopoietic stem cell transplantation using single UM171-expanded cord blood: a single-arm, phase 1–2 safety and feasibility study. Lancet Haematol. 2020;7(2):e134-45. 31704264 10.1016/S2352-3026(19)30202-9

[19] Horwitz ME, Stiff PJ, Cutler C, Brunstein C, Hanna R, Maziarz RT, et al. Omidubicel vs standard myeloablative umbilical cord blood transplantation: results of a phase 3 randomized study. Blood. 2021;138(16):1429–40. 34157093 10.1182/blood.2021011719 PMC9710469

[20] FDA approves cord-blood therapy. Nat Biotechnol. 2023;41(5):589. 10.1038/s41587-023-01808-6 37193840

[21] Wilson A, Trumpp A. Bone-marrow haematopoietic-stem-cell niches. Nat Rev Immunol. 2006;6(2):93–106. 16491134 10.1038/nri1779

[22] Friedenstein AJ, Chailakhyan RK, Latsinik NV, Panasyuk AF, Keiliss-Borok IV. Stromal cells responsible for transferring the microenvironment of the hemopoietic tissues. Transplantation. 1974;17(4):331–40. 4150881 10.1097/00007890-197404000-00001

[23] McNiece IK, Harrington J, Turney J, Kellner J, Shpall EJ. Ex vivo expansion of cord blood mononuclear cells on mesenchymal stem cells. Cytotherapy. 2004;6(4):311–7. 16146883 10.1080/14653240410004871

[24] Walenda T, Bork S, Horn P, Wein F, Saffrich R, Diehlmann A, et al. Co-culture with mesenchymal stromal cells increases proliferation and maintenance of haematopoietic progenitor cells. J Cell Mol Med. 2010;14(1–2):337. 19432817 10.1111/j.1582-4934.2009.00776.x PMC3837622

[25] Alakel N, Jing D, Muller K, Bornhauser M, Ehninger G, Ordemann R. Direct contact with mesenchymal stromal cells affects migratory behavior and gene expression profile of CD133 + hematopoietic stem cells during ex vivo expansion. Exp Hematol. 2009;37(4):504–13. 19216019 10.1016/j.exphem.2008.12.005

[26] Jing D, Fonseca AV, Alakel N, Fierro FA, Muller K, Bornhauser M, et al. Hematopoietic stem cells in co-culture with mesenchymal stromal cells - modeling the niche compartments in vitro. Haematologica. 2010;95(4):542–50. 20145267 10.3324/haematol.2009.010736 PMC2857183

[27] Corselli M, Chin CJ, Parekh C, Sahaghian A, Wang W, Ge S, et al. Perivascular support of human hematopoietic stem/progenitor cells. Blood. 2013;121(15):2891–901. 23412095 10.1182/blood-2012-08-451864 PMC3707421

[28] Blank U, Karlsson G, Karlsson S. Signaling pathways governing stem-cell fate. Blood. 2008;111(2):492–503. 17914027 10.1182/blood-2007-07-075168

[29] Aqmasheh S, Shamsasanjan K, Akbarzadehlaleh P, Sarvar DP, Timari H. Effects of mesenchymal stem cell derivatives on hematopoiesis and hematopoietic stem cells. Adv Pharm Bull. 2017;7(2):165. 28761818 10.15171/apb.2017.021 PMC5527230

[30] de Lima M, McNiece I, Robinson SN, Munsell M, Eapen M, Horowitz M, et al. Cord-blood engraftment with ex vivo mesenchymal-cell coculture. N Engl J Med. 2012;367(24):2305–15. 10.1056/NEJMoa1207285. 23234514 10.1056/NEJMoa1207285 PMC3805360

[31] Bieback K, FERNANDEZ-MUÑOZ B, PATI S. Gaps in the knowledge of human platelet lysate as a cell culture supplement for cell therapy: a joint publication from the AABB and the international society for cell & gene therapy. Cytotherapy. 2019;21(9):911–24. 31307904 10.1016/j.jcyt.2019.06.006

[32] Cherian DS, Bhuvan T, Meagher L, Heng TSP. Biological considerations in scaling up therapeutic cell manufacturing. Front Pharmacol. 2020;11:654. 32528277 10.3389/fphar.2020.00654 PMC7247829

[33] Sotiropoulou PA, Perez SA, Salagianni M, Baxevanis CN, Papamichail M. Characterization of the optimal culture conditions for clinical scale production of human mesenchymal stem cells. Stem Cells. 2006;24(2):462–71. 10.1634/stemcells.2004-0331. 16109759 10.1634/stemcells.2004-0331

[34] Sundin M, Ringdén O, Sundberg B, Nava S, Götherström C, le Blanc K. No alloantibodies against mesenchymal stromal cells, but presence of anti-fetal calf serum antibodies, after transplantation in allogeneic hematopoietic stem cell recipients. Haematologica. 2007;92(9):1208–15. 17666368 10.3324/haematol.11446

[35] n der Valk J, Brunner D, De Smet K, Fex Svenningsen Å, Honegger P, Knudsen LE, et al. Optimization of chemically defined cell culture media – Replacing fetal bovine serum in mammalian in vitro methods. Toxicol in Vitro. 2010;24(4):1053–63. 20362047 10.1016/j.tiv.2010.03.016

[36] Tancharoen W, Aungsuchawan S, Pothacharoen P, Bumroongkit K, Puaninta C, Pangjaidee N, et al. Human platelet lysate as an alternative to fetal bovine serum for culture and endothelial differentiation of human amniotic fluid mesenchymal stem cells. Mol Med Rep. 2019;19(6):5123–32. 10.3892/mmr.2019.10182/abstract. 31059024 10.3892/mmr.2019.10182 PMC6522963

[37] Guiotto M, Raffoul W, Hart AM, Riehle MO, Di Summa PG. Human platelet lysate to substitute fetal bovine serum in hMSC expansion for translational applications: a systematic review. J Transl Med. 2020;18(1):1–14. 10.1186/s12967-020-02489-4. 32933520 10.1186/s12967-020-02489-4 PMC7493356

[38] Du P, Tao X, Liu K, Lin J, Shi Y, Park K, et al. Human platelet lysate (hPL) alters the lineage commitment and paracrine functions of human mesenchymal stem cells via mitochondrial metabolism. Appl Mater Today. 2022;26:101264.

[39] Oikonomopoulos A, Van Deen WK, Manansala AR, Lacey PN, Tomakili TA, Ziman A, et al. Optimization of human mesenchymal stem cell manufacturing: the effects of animal/xeno-free media. Sci Rep. 2015;5(1):1–11. 10.1038/srep16570 PMC4643287 26564250

[40] Bucar S, Branco AD, de Mata M, Milhano MF, Caramalho JC, Cabral Í. JMS, Influence of the mesenchymal stromal cell source on the hematopoietic supportive capacity of umbilical cord blood-derived CD34+-enriched cells. Stem Cell Res Ther. 2021;12(1):1–16. 10.1186/s13287-021-02474-8. 34256848 10.1186/s13287-021-02474-8 PMC8278708

[41] dos Santos F, Andrade PZ, Boura JS, Abecasis MM, da Silva CL, Cabral JMS. Ex vivo expansion of human mesenchymal stem cells: a more effective cell proliferation kinetics and metabolism under hypoxia. J Cell Physiol. 2009. 10.1002/jcp.21987. 10.1002/jcp.21987 20020504

[42] Dominici M, Le Blanc K, Mueller I, Slaper-Cortenbach I, Marini FC, Krause DS, et al. Minimal criteria for defining multipotent mesenchymal stromal cells. The international society for cellular therapy position statement. Cytotherapy. 2006;8(4):315–7. 16923606 10.1080/14653240600855905

[43] Blighe K, Lewis M, Lun A. Package ‘PCAtools’ Title PCAtools: everything principal components analysis. 2020. https://github.com/kevinblighe/PCAtools

[44] Korotkevich G, Sukhov V, Budin N, Shpak B, Artyomov MN, Sergushichev A. Fast gene set enrichment analysis. BioRxiv. 2021. 10.1101/060012v3.

[45] Liberzon A, Birger C, Thorvaldsdóttir H, Ghandi M, Mesirov JP, Tamayo P. The Molecular Signatures Database (MSigDB) hallmark gene set collection. Cell Syst. 2015;1(6):417. 26771021 10.1016/j.cels.2015.12.004 PMC4707969

[46] Yu G, Wang LG, Han Y, He QY. ClusterProfiler. An R package for comparing biological themes among gene clusters. OMICS. 2012;16(5):284–7. 10.1089/omi.2011.0118. 22455463 10.1089/omi.2011.0118 PMC3339379

[47] Kuleshov MV, Jones MR, Rouillard AD, Fernandez NF, Duan Q, Wang Z, et al. Enrichr: a comprehensive gene set enrichment analysis web server 2016 update. Nucleic Acids Res. 2016;44(Web server issue):W90. 27141961 10.1093/nar/gkw377 PMC4987924

[48] Tormin A, Li O, Brune JC, Walsh S, Schütz B, Ehinger M, et al. CD146 expression on primary nonhematopoietic bone marrow stem cells is correlated with in situ localization. Blood. 2011;117(19):5067. 21415267 10.1182/blood-2010-08-304287 PMC3109533

[49] Tormin A, Li O, Brune JC, Walsh S, Ehinger M, Ditzel N, et al. Human primary CD271+/CD45–/CD146–/Low and CD271+/CD45–/CD146 + bone marrow cells are developmentally closely-related stroma stem cells with similar functional properties but different in-situ localization. Blood. 2010;116(21):2594–2594.

[50] Palombella S, Perucca Orfei C, Castellini G, Gianola S, Lopa S, Mastrogiacomo M, et al. Systematic review and meta-analysis on the use of human platelet lysate for mesenchymal stem cell cultures: comparison with fetal bovine serum and considerations on the production protocol. Stem Cell Res Ther. 2022;13(1):1–31. 10.1186/s13287-022-02815-1. 35379348 10.1186/s13287-022-02815-1 PMC8981660

[51] Fernandez-Rebollo E, Mentrup B, Ebert R, Franzen J, Abagnale G, Sieben T, et al. Human platelet lysate versus fetal calf serum: these supplements do not select for different mesenchymal stromal cells. Sci Rep. 2017;7(1):1–8. 28698620 10.1038/s41598-017-05207-1 PMC5506010

[52] Hagmann S, Moradi B, Frank S, Dreher T, Kämmerer PW, Richter W, et al. Different culture media affect growth characteristics, surface marker distribution and chondrogenic differentiation of human bone marrow-derived mesenchymal stromal cells. BMC Musculoskelet Disord. 2013;14(1):1–11. 10.1186/1471-2474-14-223. 23898974 10.1186/1471-2474-14-223 PMC3734101

[53] Winkel A, Jaimes Y, Melzer C, Dillschneider P, Hartwig H, Stiesch M, et al. Cell culture media notably influence properties of human mesenchymal stroma/stem-like cells from different tissues. Cytotherapy. 2020;22(11):653–68. 32855067 10.1016/j.jcyt.2020.07.005

[54] Kim K, Thorp H, Bou-Ghannam S, Grainger DW, Okano T. Stable cell adhesion affects mesenchymal stem cell sheet fabrication: effects of fetal bovine serum and human platelet lysate. J Tissue Eng Regen Med. 2020;14(5):741–53. 10.1002/term.3037. 32212212 10.1002/term.3037

[55] Cheng NC, Tu YK, Lee NH, Young TH. Influence of human platelet lysate on extracellular matrix deposition and cellular characteristics in adipose-derived stem cell sheets. Front Cell Dev Biol. 2020;8:558354. 33195191 10.3389/fcell.2020.558354 PMC7642065

[56] Alakel N, Jing D, Bornhaeuser M, Ehninger G, Ordemann R. CD133 + purified hematopoietic stem cells in co-culture with mesenchymal stromal cells - the cell to cell contact matters. Blood. 2007;110(11):1421–1421. 10.1182/blood.V110.11.1421.1421.

[57] Wagner W, Wein F, Roderburg C, Benes V, Diehlmann A, Krause U, et al. Adhesion of hematopoietic progenitor cells to human mesenchymal stromal cells as a model for interaction between stem cells and their niche. Blood. 2006;108(11):1399–1399. 10.1182/blood.V108.11.1399.1399.

[58] Mishima S, Nagai A, Abdullah S, Matsuda C, Taketani T, Kumakura S, et al. Effective ex vivo expansion of hematopoietic stem cells using osteoblast-differentiated mesenchymal stem cells is CXCL12 dependent. Eur J Haematol. 2010;84(6):538–46. 10.1111/j.1600-0609.2010.01419.x. 20088916 10.1111/j.1600-0609.2010.01419.x

[59] Majumdar MK, Thiede MA, Haynesworth SE, Bruder SP, Gerson SL. Human marrow-derived mesenchymal stem cells (MSCs) express hematopoietic cytokines and support long-term hematopoiesis when differentiated toward stromal and osteogenic lineages. J Hematother Stem Cell Res. 2004;9(6):841–8. 10.1089/152581600750062264. 10.1089/152581600750062264 11177595

[60] Wilkinson AC, Igarashi KJ, Nakauchi H. Haematopoietic stem cell self-renewal in vivo and ex vivo. Nat Reiews Genet. 2020;21(9):541–54. 10.1038/s41576-020-0241-0 PMC7894993 32467607

[61] Wuchter P, Diehlmann A, Klüter H. Closer to nature: the role of MSCs in recreating the microenvironment of the hematopoietic stem cell niche in vitro. Transfus Med Hemother. 2022;49(4):258–67. 36159960 10.1159/000520932 PMC9421702

[62] Medvedev N, Yurievna Zavalishina S, Viktorovna Vorobieva N, Xuan J, Liu Y, Liu J, et al. New insights into hematopoietic stem cell expansion to stimulate repopulation of the adult blood system for transplantation. Life. 2022;12(5):716. 35629383 10.3390/life12050716 PMC9146250

[63] Walasek MA, van Os R, de Haan G. Hematopoietic stem cell expansion: challenges and opportunities. Ann N Y Acad Sci. 2012;1266(1):138–50. 10.1111/j.1749-6632.2012.06549.x. 22901265 10.1111/j.1749-6632.2012.06549.x

[64] Méndez-Ferrer S, Bonnet D, Steensma DP, Hasserjian RP, Ghobrial IM, Gribben JG, et al. Bone marrow niches in haematological malignancies. Nat Rev Cancer. 2020;20(5):285–98. 32112045 10.1038/s41568-020-0245-2 PMC9912977

[65] Fröbel J, Landspersky T, Percin G, Schreck C, Rahmig S, Ori A, et al. The hematopoietic bone marrow niche ecosystem. Front Cell Dev Biol. 2021;9:705410. 34368155 10.3389/fcell.2021.705410 PMC8339972

[66] Li T, Wu Y. Paracrine molecules of mesenchymal stem cells for hematopoietic stem cell niche. Bone Marrow Res. 2011;2011:1–8. 10.1155/2011/353878 PMC3196250 22046560

[67] Rizo A, Vellenga E, de Haan G, Schuringa JJ. Signaling pathways in self-renewing hematopoietic and leukemic stem cells: do all stem cells need a niche? Hum Mol Genet. 2006;15(suppl_2):R210-9. 10.1093/hmg/ddl175. 16987886 10.1093/hmg/ddl175

[68] Kulkarni R, Kale V. Physiological cues involved in the regulation of adhesion mechanisms in hematopoietic stem cell fate decision. Front Cell Dev Biol. 2020;8:552147. 10.3389/fcell.2020.00611 PMC7366553 32754597

[69] Hurwitz SN, Jung SK, Kurre P. Hematopoietic stem and progenitor cell signaling in the niche. Leukemia. 2020;34(12):3136–48. 33077865 10.1038/s41375-020-01062-8

[70] Stier S, Ko Y, Forkert R, Lutz C, Neuhaus T, Grünewald E, et al. Osteopontin is a hematopoietic stem cell niche component that negatively regulates stem cell pool size. J Exp Med. 2005;201(11):1781–91. 10.1084/jem.20041992. 15928197 10.1084/jem.20041992 PMC2213260

[71] Chin CJ, Li S, Corselli M, Casero D, Zhu Y, He C, Bin, et al. Transcriptionally and functionally distinct mesenchymal subpopulations are generated from human pluripotent stem cells. Stem Cell Rep. 2018;10(2):436–46. 10.1016/j.stemcr.2017.12.005 PMC5830911 29307583

[72] Vukicevic S, Grgurevic L. BMP-6 and mesenchymal stem cell differentiation. Cytokine Growth Factor Rev. 2009;20(5–6):441–8. 19900832 10.1016/j.cytogfr.2009.10.020

[73] Bhatia M, Bonnet D, Wu D, Murdoch B, Wrana J, Gallacher L, et al. Bone morphogenetic proteins regulate the developmental program of human hematopoietic stem cells. J Exp Med. 1999;189(7):1139–48. 10190905 10.1084/jem.189.7.1139 PMC2193014

[74] Kieslinger M, Hiechinger S, Dobreva G, Consalez GG, Grosschedl R. Early B cell factor 2 regulates hematopoietic stem cell homeostasis in a cell-nonautonomous manner. Cell Stem Cell. 2010;7(4):496–507. 20887955 10.1016/j.stem.2010.07.015

[75] Paciejewska MM, Maijenburg MW, Gilissen C, Kleijer M, Vermeul K, Weijer K, et al. Different balance of Wnt signaling in adult and fetal bone marrow-derived mesenchymal stromal cells. Stem Cells Dev. 2016;25(12):934–47. 10.1089/scd.2015.0263. 27154244 10.1089/scd.2015.0263

[76] n den Berg DJ, Sharma AK, Bruno E, Hoffman R. Role of members of the Wnt gene family in human hematopoiesis. Blood. 1998;92(9):3189–202. 9787155

[77] Jridi I, Pike-Overzet K, Cante-Barrett K, Staal FJ. Wnt signaling in lymphopoiesis and hematopoiesis. J Stem Cell Res (Overl Park). 2020;1(2):1–27.

[78] Smith JNP, Calvi LM. Concise review: current concepts in bone marrow microenvironmental regulation of hematopoietic stem and progenitor cells. Stem Cells. 2013;31(6):1044–50. 10.1002/stem.1370. 23509002 10.1002/stem.1370 PMC3664122

[79] O’Hagan-Wong K, Nadeau S, Carrier-Leclerc A, Apablaza F, Hamdy R, Shum-Tim D, et al. Increased IL-6 secretion by aged human mesenchymal stromal cells disrupts hematopoietic stem and progenitor cells’ homeostasis. Oncotarget. 2016;7(12):13285. 26934440 10.18632/oncotarget.7690 PMC4924641

[80] Yahata T, Ibrahim AA, Muguruma Y, Eren M, Shaffer AM, Watanabe N, et al. TGF-β–induced intracellular PAI-1 is responsible for retaining hematopoietic stem cells in the niche. Blood. 2017;130(21):2283–94. 10.1182/blood-2017-02-767384. 28821477 10.1182/blood-2017-02-767384 PMC5701521

[81] Russell Taichman MS, Emerson SG, Taichman RS. The role of osteoblasts in the hematopoietic microenvironment. Stem Cells. 1998;16(1):7–15. 10.1002/stem.160007. 9474743 10.1002/stem.160007

[82] Bromberg O, Frisch BJ, Weber JM, Porter RL, Civitelli R, Calvi LM. Osteoblastic N-cadherin is not required for microenvironmental support and regulation of hematopoietic stem and progenitor cells. Blood. 2012;120(2):303–13. 10.1182/blood-2011-09-377853. 22596259 10.1182/blood-2011-09-377853 PMC3398755

[83] Chitteti BR, Kobayashi M, Cheng Y, Zhang H, Poteat BA, Broxmeyer HE, et al. CD166 regulates human and murine hematopoietic stem cells and the hematopoietic niche. Blood. 2014;124(4):519–29. 10.1182/blood-2014-03-565721. 24740813 10.1182/blood-2014-03-565721 PMC4110658

[84] De Grandis M, Lhoumeau AC, Mancini SJC, Aurrand-Lions M. Adhesion receptors involved in HSC and early-B cell interactions with bone marrow microenvironment. Cell Mol Life Sci. 2015;73(4):687–703. 10.1007/s00018-015-2064-2. 10.1007/s00018-015-2064-2 PMC11108274 26495446

[85] Arcangeli ML, Frontera V, Bardin F, Obrados E, Adams S, Chabannon C, et al. JAM-B regulates maintenance of hematopoietic stem cells in the bone marrow. Blood. 2011;118(17):4609–19. 10.1182/blood-2010-12-323972. 21868569 10.1182/blood-2010-12-323972

[86] Duryagina R, Thieme S, Anastassiadis K, Werner C, Schneider S, Wobus M, et al. Overexpression of jagged-1 and its intracellular domain in human mesenchymal stromal cells differentially affect the interaction with hematopoietic stem and progenitor cells. Stem Cells Dev. 2013;22(20):2736–50. 10.1089/scd.2012.0638. 23758219 10.1089/scd.2012.0638

