# Supplementary material for: Differential effects of fetal bovine serum and human platelet lysate on mesenchymal stromal cell-mediated support of hematopoietic stem/progenitor cells: a functional and transcriptomic analysis
Source: Stem Cell Res Ther. 2025 Nov 29;17:6. doi: 10.1186/s13287-025-04835-z (PMC12771711; doi:10.1186/s13287-025-04835-z)
Supplement: Supplementary file 1 — Supplementary Material 1. Supplementary figures and tables. Composed of supplementary Tables 1 and supplementary Figs. 1–4. [file 13287_2025_4835_MOESM1_ESM.pptx]

## Slide 1
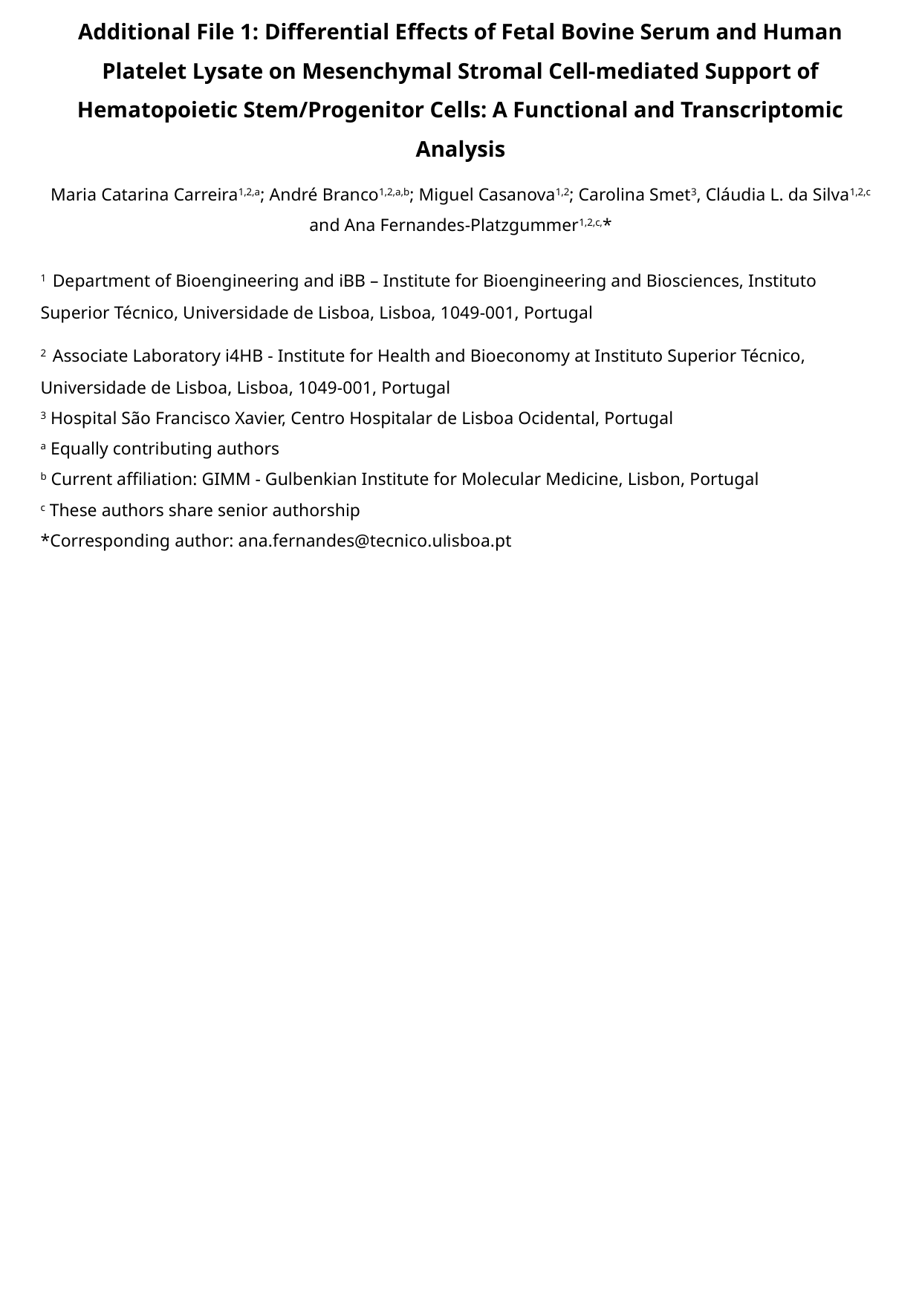

Additional File 1: Differential Effects of Fetal Bovine Serum and Human Platelet Lysate on Mesenchymal Stromal Cell-mediated Support of Hematopoietic Stem/Progenitor Cells: A Functional and Transcriptomic Analysis
Maria Catarina Carreira1,2,a; André Branco1,2,a,b; Miguel Casanova1,2; Carolina Smet3, Cláudia L. da Silva1,2,c and Ana Fernandes-Platzgummer1,2,c,*
1 Department of Bioengineering and iBB – Institute for Bioengineering and Biosciences, Instituto Superior Técnico, Universidade de Lisboa, Lisboa, 1049-001, Portugal
2 Associate Laboratory i4HB - Institute for Health and Bioeconomy at Instituto Superior Técnico, Universidade de Lisboa, Lisboa, 1049-001, Portugal
3 Hospital São Francisco Xavier, Centro Hospitalar de Lisboa Ocidental, Portugal
a Equally contributing authors
b Current affiliation: GIMM - Gulbenkian Institute for Molecular Medicine, Lisbon, Portugal
c These authors share senior authorship
*Corresponding author: ana.fernandes@tecnico.ulisboa.pt

## Slide 2
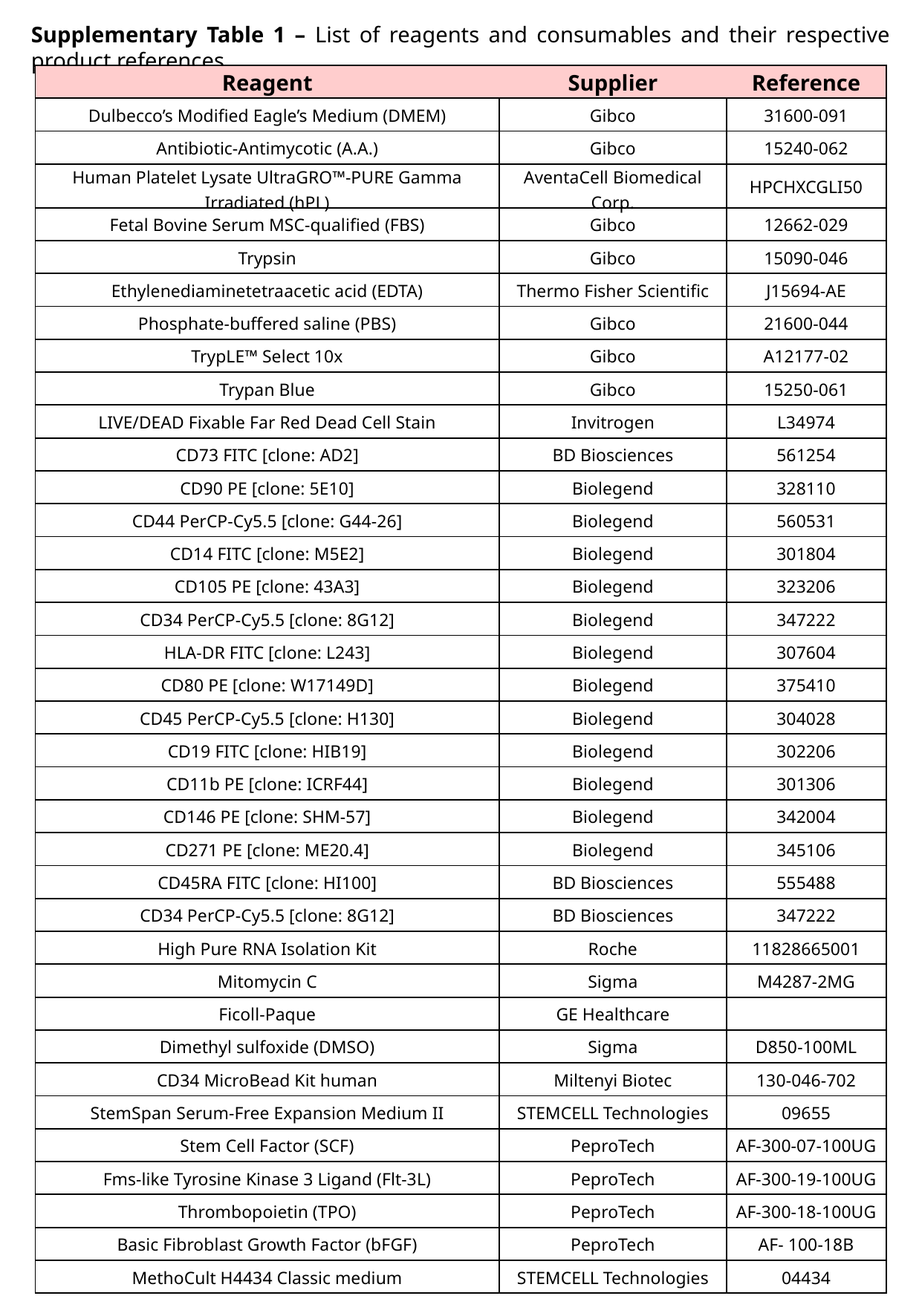

Supplementary Table 1 – List of reagents and consumables and their respective product references.
| Reagent | Supplier | Reference |
| --- | --- | --- |
| Dulbecco’s Modified Eagle’s Medium (DMEM) | Gibco | 31600-091 |
| Antibiotic-Antimycotic (A.A.) | Gibco | 15240-062 |
| Human Platelet Lysate UltraGRO™-PURE Gamma Irradiated (hPL) | AventaCell Biomedical Corp. | HPCHXCGLI50 |
| Fetal Bovine Serum MSC-qualified (FBS) | Gibco | 12662-029 |
| Trypsin | Gibco | 15090-046 |
| Ethylenediaminetetraacetic acid (EDTA) | Thermo Fisher Scientific | J15694-AE |
| Phosphate-buffered saline (PBS) | Gibco | 21600-044 |
| TrypLE™ Select 10x | Gibco | A12177-02 |
| Trypan Blue | Gibco | 15250-061 |
| LIVE/DEAD Fixable Far Red Dead Cell Stain | Invitrogen | L34974 |
| CD73 FITC [clone: AD2] | BD Biosciences | 561254 |
| CD90 PE [clone: 5E10] | Biolegend | 328110 |
| CD44 PerCP-Cy5.5 [clone: G44-26] | Biolegend | 560531 |
| CD14 FITC [clone: M5E2] | Biolegend | 301804 |
| CD105 PE [clone: 43A3] | Biolegend | 323206 |
| CD34 PerCP-Cy5.5 [clone: 8G12] | Biolegend | 347222 |
| HLA-DR FITC [clone: L243] | Biolegend | 307604 |
| CD80 PE [clone: W17149D] | Biolegend | 375410 |
| CD45 PerCP-Cy5.5 [clone: H130] | Biolegend | 304028 |
| CD19 FITC [clone: HIB19] | Biolegend | 302206 |
| CD11b PE [clone: ICRF44] | Biolegend | 301306 |
| CD146 PE [clone: SHM-57] | Biolegend | 342004 |
| CD271 PE [clone: ME20.4] | Biolegend | 345106 |
| CD45RA FITC [clone: HI100] | BD Biosciences | 555488 |
| CD34 PerCP-Cy5.5 [clone: 8G12] | BD Biosciences | 347222 |
| High Pure RNA Isolation Kit | Roche | 11828665001 |
| Mitomycin C | Sigma | M4287-2MG |
| Ficoll-Paque | GE Healthcare | |
| Dimethyl sulfoxide (DMSO) | Sigma | D850-100ML |
| CD34 MicroBead Kit human | Miltenyi Biotec | 130-046-702 |
| StemSpan Serum-Free Expansion Medium II | STEMCELL Technologies | 09655 |
| Stem Cell Factor (SCF) | PeproTech | AF-300-07-100UG |
| Fms-like Tyrosine Kinase 3 Ligand (Flt-3L) | PeproTech | AF-300-19-100UG |
| Thrombopoietin (TPO) | PeproTech | AF-300-18-100UG |
| Basic Fibroblast Growth Factor (bFGF) | PeproTech | AF- 100-18B |
| MethoCult H4434 Classic medium | STEMCELL Technologies | 04434 |

## Slide 3
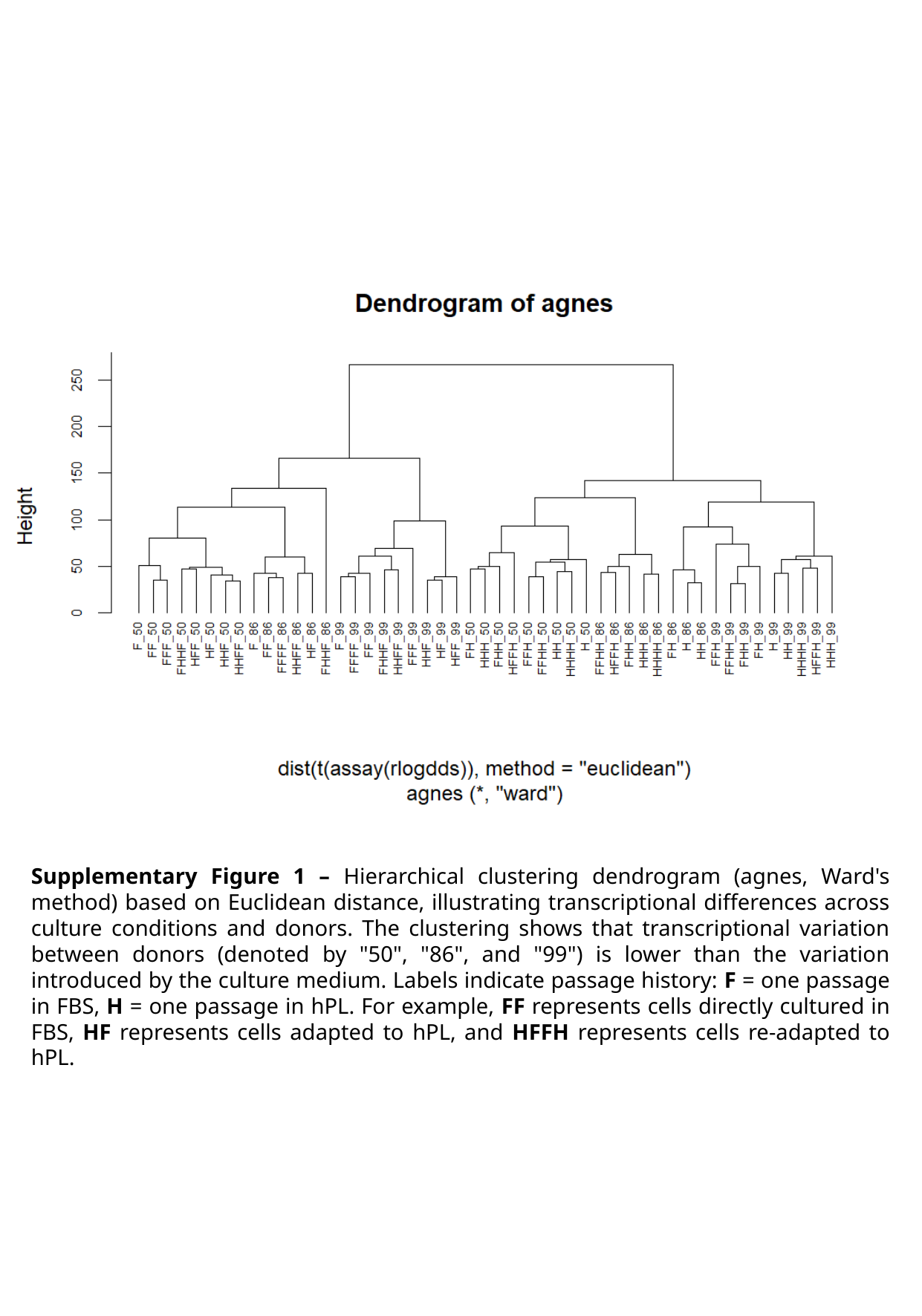

Supplementary Figure 1 – Hierarchical clustering dendrogram (agnes, Ward's method) based on Euclidean distance, illustrating transcriptional differences across culture conditions and donors. The clustering shows that transcriptional variation between donors (denoted by "50", "86", and "99") is lower than the variation introduced by the culture medium. Labels indicate passage history: F = one passage in FBS, H = one passage in hPL. For example, FF represents cells directly cultured in FBS, HF represents cells adapted to hPL, and HFFH represents cells re-adapted to hPL.

## Slide 4
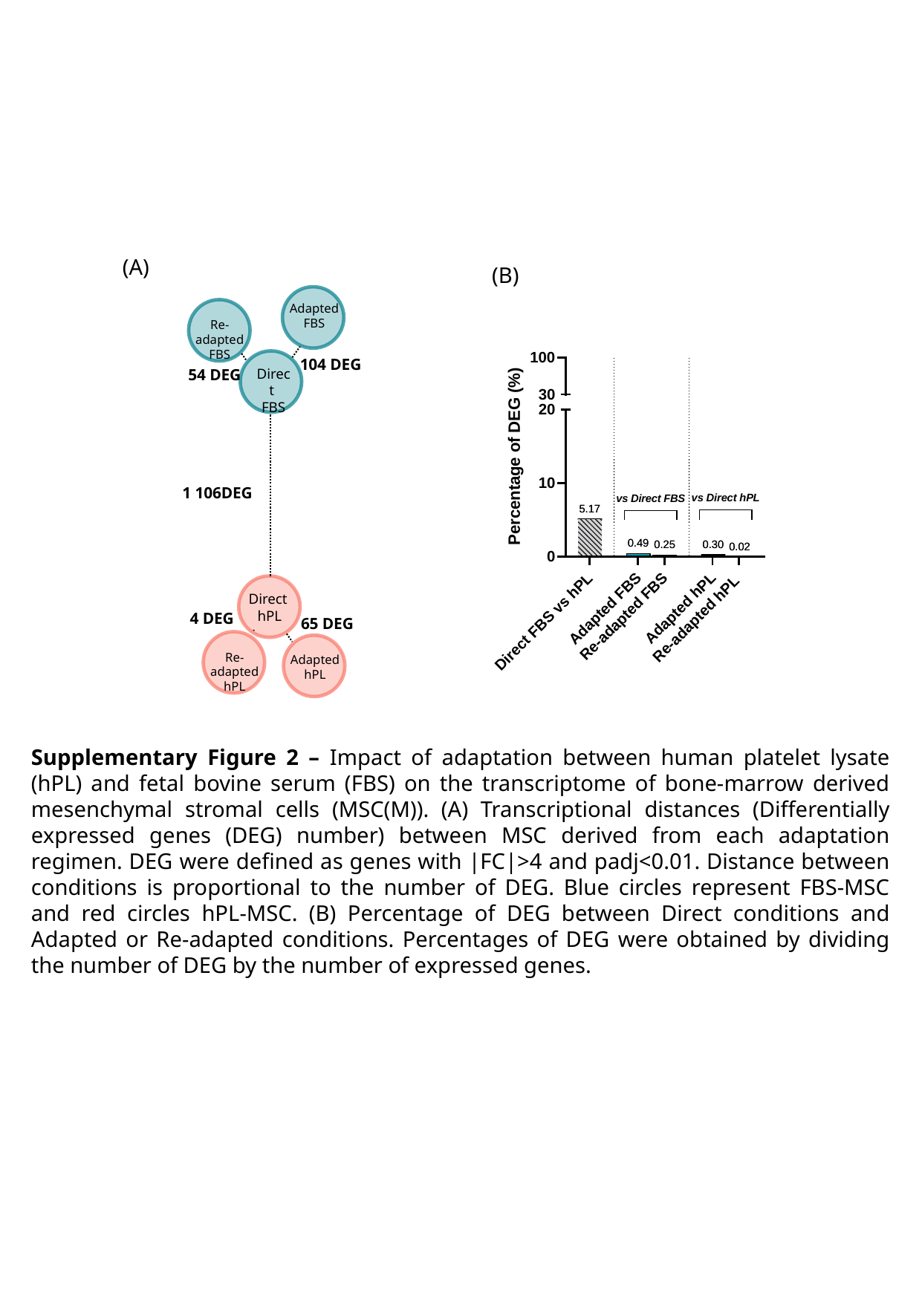

(A)
(B)
Adapted FBS
Re-adapted FBS
104 DEG
54 DEG
Direct
FBS
1 106DEG
Direct
hPL
4 DEG
65 DEG
Re-adapted hPL
Adapted hPL
Supplementary Figure 2 – Impact of adaptation between human platelet lysate (hPL) and fetal bovine serum (FBS) on the transcriptome of bone-marrow derived mesenchymal stromal cells (MSC(M)). (A) Transcriptional distances (Differentially expressed genes (DEG) number) between MSC derived from each adaptation regimen. DEG were defined as genes with |FC|>4 and padj<0.01. Distance between conditions is proportional to the number of DEG. Blue circles represent FBS-MSC and red circles hPL-MSC. (B) Percentage of DEG between Direct conditions and Adapted or Re-adapted conditions. Percentages of DEG were obtained by dividing the number of DEG by the number of expressed genes.

## Slide 5
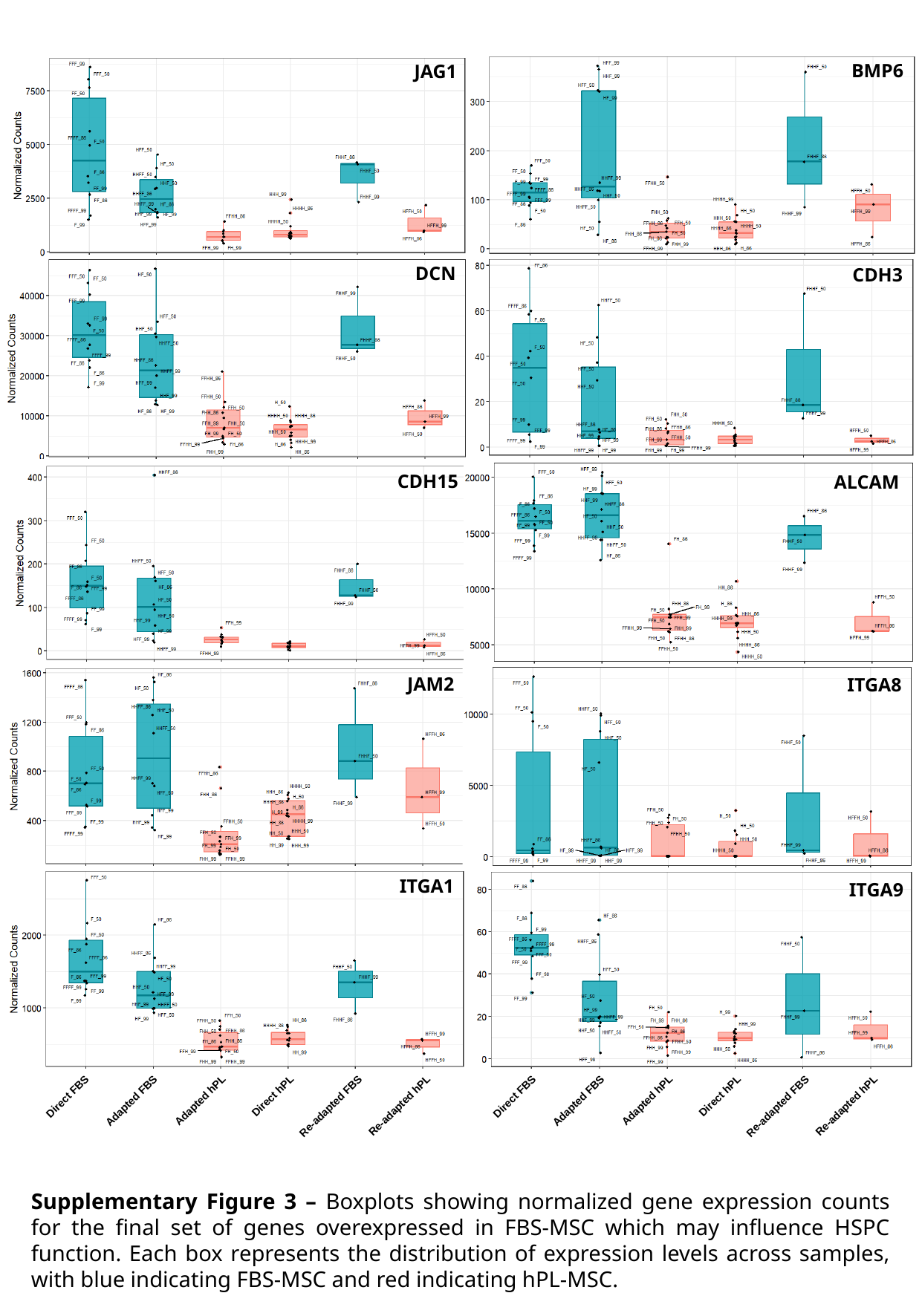

BMP6
JAG1
DCN
CDH3
CDH15
ALCAM
JAM2
ITGA8
ITGA1
ITGA9
Direct FBS
Adapted FBS
Adapted hPL
Direct hPL
Re-adapted FBS
Re-adapted hPL
Direct FBS
Adapted FBS
Adapted hPL
Direct hPL
Re-adapted FBS
Re-adapted hPL
Supplementary Figure 3 – Boxplots showing normalized gene expression counts for the final set of genes overexpressed in FBS-MSC which may influence HSPC function. Each box represents the distribution of expression levels across samples, with blue indicating FBS-MSC and red indicating hPL-MSC.

## Slide 6
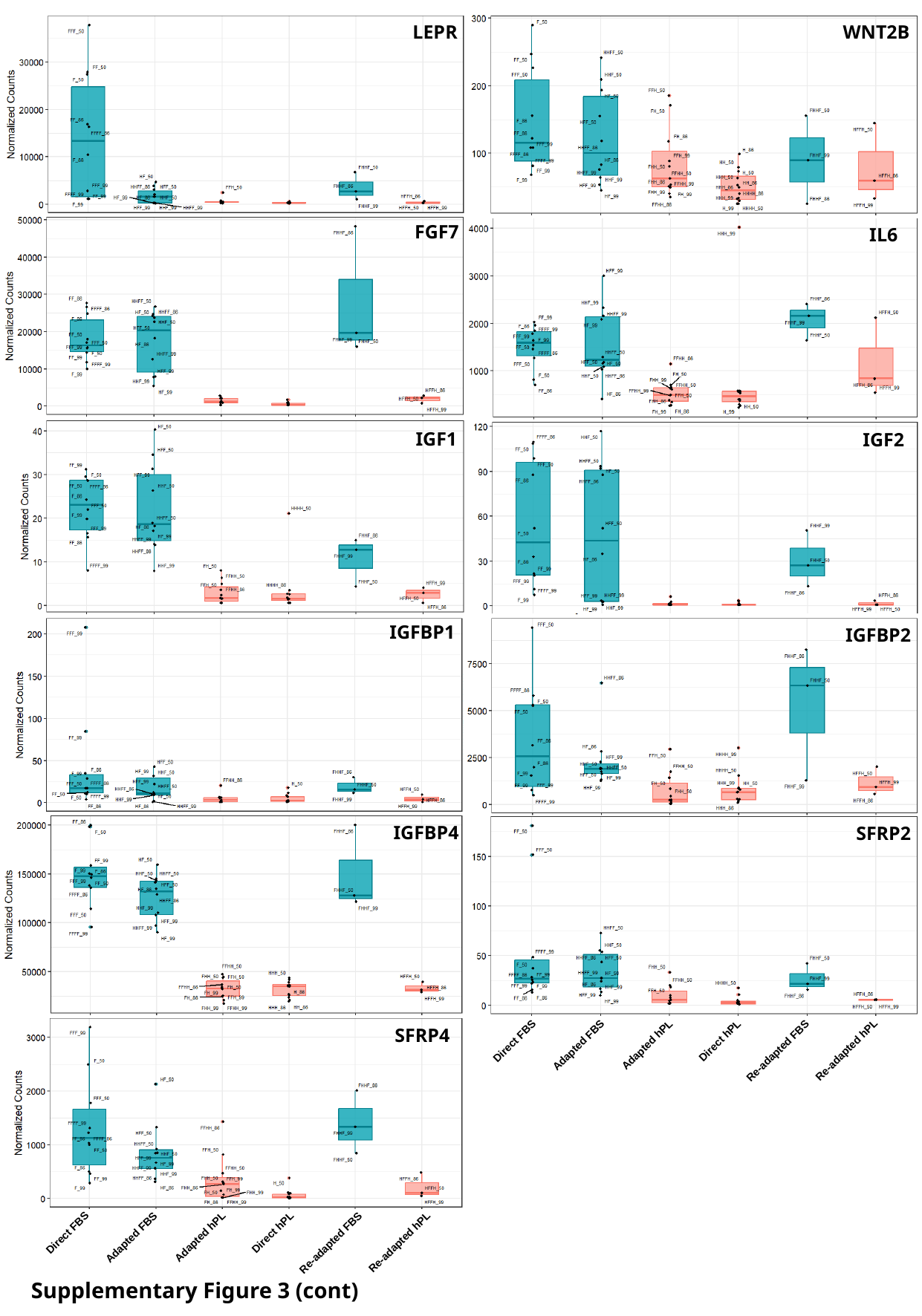

LEPR
WNT2B
FGF7
IL6
IGF1
IGF2
IGFBP1
IGFBP2
IGFBP4
SFRP2
SFRP4
Direct FBS
Adapted FBS
Adapted hPL
Direct hPL
Re-adapted FBS
Re-adapted hPL
Direct FBS
Adapted FBS
Adapted hPL
Direct hPL
Re-adapted FBS
Re-adapted hPL
Supplementary Figure 3 (cont)

## Slide 7
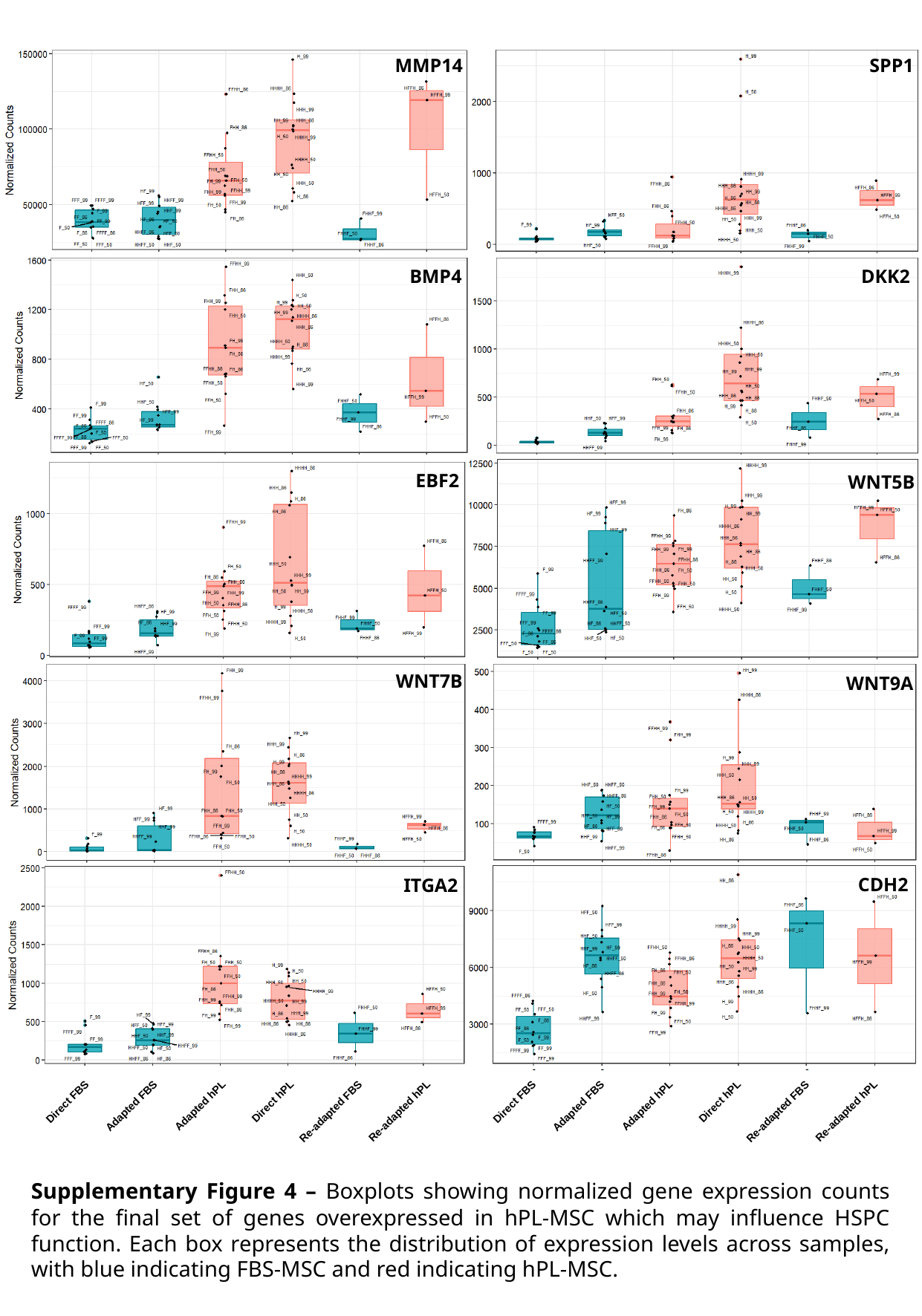

SPP1
MMP14
DKK2
BMP4
EBF2
WNT5B
WNT7B
WNT9A
CDH2
ITGA2
Direct FBS
Adapted FBS
Adapted hPL
Direct hPL
Re-adapted FBS
Re-adapted hPL
Direct FBS
Adapted FBS
Adapted hPL
Direct hPL
Re-adapted FBS
Re-adapted hPL
Supplementary Figure 4 – Boxplots showing normalized gene expression counts for the final set of genes overexpressed in hPL-MSC which may influence HSPC function. Each box represents the distribution of expression levels across samples, with blue indicating FBS-MSC and red indicating hPL-MSC.
